# Evaluating deep learning sepsis prediction models in ICUs under distribution shift: a multi-centre retrospective cohort study

**DOI:** 10.1038/s41746-026-02364-4

**Published:** 2026-03-03

**Authors:** Fanny Tranchellini, Youssef Farag, Catherine Jutzeler, Lakmal Meegahapola

**Affiliations:** 1 Department of Health Sciences and Technology, ETH Zurich, Zurich, Switzerland; 2 Swiss Institute of Bioinformatics, Lausanne, Switzerland

**Keywords:** Computational biology and bioinformatics, Diseases, Health care, Mathematics and computing, Medical research

## Abstract

Sepsis prediction models trained on ICU data often fail to generalize under external validation because of distribution shift. Prior studies have focused on direct model deployment or conventional transfer learning methods (e.g., fine-tuning), yet systematic exploration of alternative strategies remains limited. We quantify shifts across three harmonized adult ICU cohorts (HiRID, MIMIC-IV, eICU; 216,536 stays) and compare five deployment strategies: generalization, fine-tuning/retraining, target training, supervised domain adaptation (DA), and fusion-training, across multiple deep learning architectures, and four target-data regimes (none; small ≤ 8k; medium 8–32k; large ≥ 32k stays). Fine-tuning consistently underperforms, even though it has been the go-to method in literature. Retraining and fusion perform best in small and large target data regimes, while DA yields the most stable gains with medium target data, improving AUROC and normalized AUPRC over other methods. These results argue for moving beyond routine fine-tuning for sepsis prediction and selecting strategies by target-data availability and operational context.

## Introduction

Sepsis is a life-threatening disease and one of the leading causes of hospital mortality worldwide, particularly within intensive care units (ICU)^1–4^. It occurs when an infection triggers a dysregulated immune response, leading to widespread inflammation, tissue damage, and impaired organ function^4^. Sepsis is difficult to diagnose early due to its rapid progression and varied clinical presentation; however, timely detection is critical to improve patient outcomes^2–5^. Artificial intelligence (AI), particularly deep learning (DL), offers a promising approach to early detection of sepsis by identifying predictive patterns within the extensive clinical data generated in ICUs, including vital signs, laboratory results, and demographics of patients^3,6–9^. The choice of ICUs specifically was motivated by the high proportion of ICU deaths caused by sepsis^4^, coupled with the recognition that precise and timely interventions can significantly impact patient outcomes^10^. However, a key barrier to the widespread clinical adoption of AI-based predictive models is their poor generalizability between sites. While models typically perform well at their training sites, their accuracy tends to decline at external sites due to *distribution shifts*^11^. These shifts are due to variations in data characteristics, such as differences in vital sign distributions, labeling practices, patient demographics, and clinical protocols, between training (source domain) and deployment (target domain) sites^12^. This challenge was highlighted by a study that showed substantial performance deterioration of a commercial sepsis prediction model, where the model performance dropped to an AUROC of only 0.63 when externally validated in multiple hospitals in the United States, a performance far lower than what the company that developed the model quoted^13^.

Although the need to generalize across sites is widely recognized as a critical challenge, most existing sepsis prediction studies focus on two simplified deployment scenarios^1^: external validation and standard transfer learning with fine-tuning. External validation is particularly important when data from the target site are unavailable, and a model trained on source datasets must be deployed without modification. In such cases, the expectation is that the model should remain robust across different environments. Standard transfer learning approaches, on the other hand, assume only a minor distribution shift between the source and target domains and therefore rely on a small amount of target data to adapt the models to the new setting^14^. Hence, both of these approaches assume that a pre-trained model is available for deployment at the target site. However, other potentially useful strategies, such as supervised domain adaptation (DA), remain largely unexplored when source and target data are available^1,11,15^. This gap is problematic because hospitals differ substantially in their resources, data volumes, data sharing practices, and computational capabilities, which demand flexible strategies suited to specific operational contexts.

**Fig. 1:**
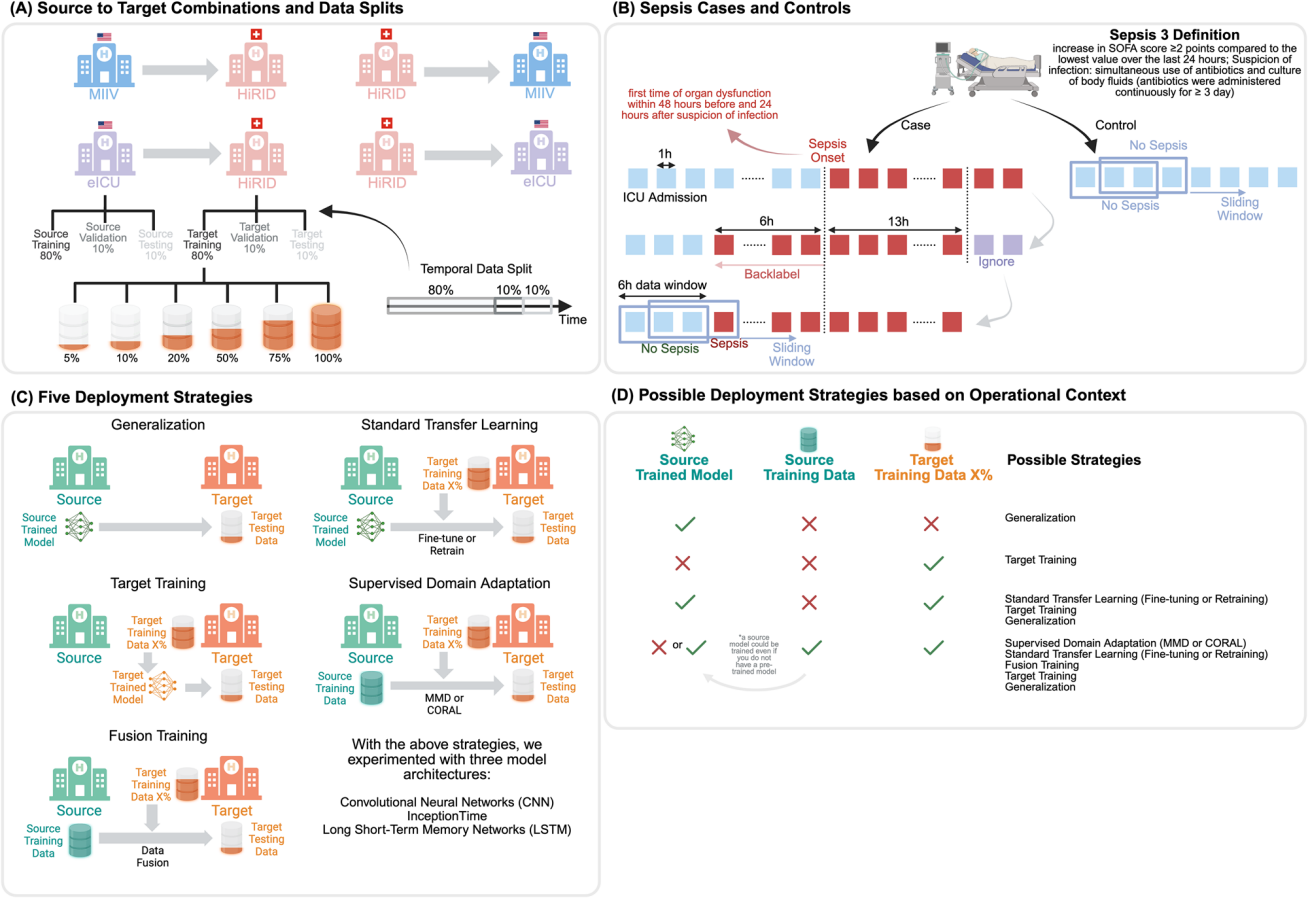
Study design and evaluation framework for cross-dataset sepsis prediction. **A** When evaluating model performance, four combinations were considered. For each dataset, we predefined training, validation, and testing datasets. Depending on whether a dataset serves as the source or target in each combination, the relevant training and validation sets were used to train models. Data were split temporally; (**B)** shows how cases and controls are identified, and how a 6-hour sliding window was used to sample data for model training. We applied a strict version of the Sepsis-3 definition to determine onset, inspired by the work by ref. ^18^; (**C)** Presents the five main strategies evaluated in this study. For each strategy, it also indicates whether data or models from the source or target domain are utilized; (**D)** illustrates several realistic operational contexts in terms of model and data availability in the source and target domains for training a model. For each scenario, possible strategies from **C** that could be applied are mentioned. This demonstrates that all five broad strategies could be useful in specific setups, making them worth comparing. For more on data harmonization and pre-processing, see Supplementary Section I.

Real-world scenarios highlight the importance of tailoring deployment strategies. For example, in a rural hospital with limited initial data, a model pre-trained at a larger clinical center may first be deployed directly (generalization) and later fine-tuned or fully retrained as local data accumulate. Eventually, with sufficient local data, it may become feasible to train a standalone model from scratch (target training). In a hospital network aiming to standardize sepsis prediction across multiple affiliated sites, data can be pooled across institutions (fusion training), while individual hospitals may also fine-tune a central model or adopt supervised domain adaptation methods to address distributional differences. At a national scale, a model trained in large urban hospitals could be deployed directly (generalization) in smaller rural facilities, with subsequent fine-tuning or retraining as more local data become available. Such large-scale initiatives could particularly benefit from methods that explicitly account for domain shifts, such as supervised domain adaptation or fusion training, when integrating highly diverse datasets. These variations in model and data availability across sites, depending on the operational context (Fig. 1**F**), create the need for systematic evaluation and comparison of deployment strategies (Fig. 1**E**), including generalization (direct use of a source-trained model without modification), standard transfer learning (fine-tuning or retraining a pre-trained model with target data), target training (training solely on target-domain data), supervised domain adaptation (explicit alignment of feature distributions using methods such as maximum mean discrepancy (MMD)^16^ or correlation alignment (CORAL)^17^), and fusion training (combining source and target datasets into a unified training set). Each of these approaches addresses distinct challenges, including data scarcity, domain mismatch, data-sharing limitations, and practical deployment constraints. However, prior studies often overlook such diverse operational contexts, rarely conduct rigorous evaluations of advanced methods like supervised domain adaptation, and seldom provide critical comparisons of strategies under realistic deployment settings^15^.

In this study, we address existing gaps by systematically evaluating the effectiveness of various model deployment strategies for sepsis prediction across heterogeneous ICU settings, providing three key contributions. First, we rigorously quantify the distribution shifts among three large ICU patient cohorts (MIMIC-IV, eICU, and HiRID) to clarify how variations in clinical practices, patient demographics, and data acquisition methods influence their statistical properties, establishing a foundation for understanding generalizability. These datasets were selected for their high temporal resolution and documentation standards, harmonized and pre-processed in accordance with published benchmarks^18^ and pipelines^19^. Second, we assess cross-domain performance by training on one ICU (source) and validating on another (target) across four pairings: HiRID-MIIV, HiRID-eICU, eICU-HiRID, and MIIV-HiRID. Finally, to mirror real-world deployments, we analyze two practical contexts, one where only a pre-trained source model is available and another granting access to source-domain data, tracing performance as progressively larger fractions of target data (5%, 10%, 20%, 50%, 75%, 100%) become available. Under these conditions, we compare five deployment options: direct generalization, fine-tuning/retraining, supervised domain adaptation (MMD/CORAL), target training, and naive fusion training. Our results indicate a hierarchy dependent on target data availability. When data are scarce (≤10%), retraining the source model proves to be the most reliable strategy, followed closely by fusion training; both methods outperform fine-tuning and direct generalization by up to 14% AUROC. At moderate data volumes (≈20–50%), supervised domain adaptation methods yield the most stable gains, producing 3–8% AUROC and 0.2–0.6 normalized AUPRC improvements over retraining and fine-tuning, while exhibiting the lowest variance. Once large target datasets (≥75%) are available, models trained wholly or partly on the target cohort (target training or fusion) match or surpass transfer learning alternatives. The benefit of any strategy is modulated by dataset similarity; for instance, eICU-trained models generalize better to HiRID than MIIV-trained ones. These findings suggest caution regarding default reliance on fine-tuning and highlight the necessity for deployment plans that adapt to domain shift, data scale, and resource constraints, guidance potentially applicable to a broad range of critical care applications.

## Results

The results are organized into three key sections: the existence and significance of distribution shifts between sites, the generalizability of deep learning models across diverse ICU cohorts, and the optimal strategies for deploying these models in target domains. The results are primarily reported for AUROC and normalized AUPRC. These metrics are threshold-independent, allowing for comparison of the overall performance of a model across all possible thresholds. These are also the most commonly reported metrics in studies with a similar focus/approach^11,18,20–23^.

### Existence and significance of distribution shifts between sites

Our analysis confirmed that covariate distribution shifts are common and often large between ICU datasets. The Kolmogorov-Smirnov (K-S) tests revealed numerous features with significantly different distributions across the three pairwise dataset comparisons (HiRID vs. MIIV, HiRID vs. eICU, and MIIV vs. eICU). Of the 48 dynamic characteristics examined, between 18 and 29 characteristics (37-60%) showed highly significant differences (*p* < 0.01) in all four summary statistics when comparing two sites. In other words, each pair of ICU cohorts had dozens of variables that did not follow the same patterns. The HiRID-MIIV pairing had 18 features significantly different in mean/variance/min/max (with an additional three features differing in three of those stats), HiRID-eICU had 19 (plus five with partial differences), and MIIV-eICU showed the most with 29 (plus eight partial). These statistical tests underscore that if one trains a model on the data of an ICU, many input variables will behave differently in the setting of another ICU, violating the assumptions of the model.

**Fig. 2:**
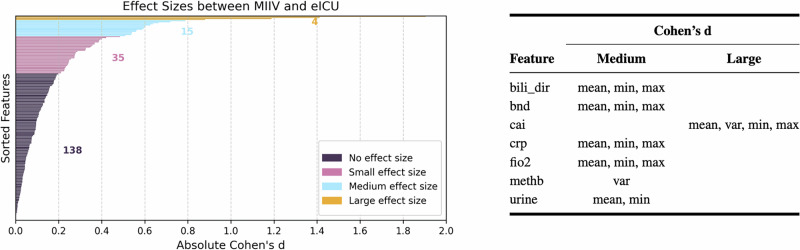
Representation of Cohen’s d analysis of American (MIIV-eICU) dataset pairing. Left: Visualization of the number of features into effect size categories based on absolute Cohen’s d. Right: Classification of features into medium and large effect size categories.

**Fig. 3:**
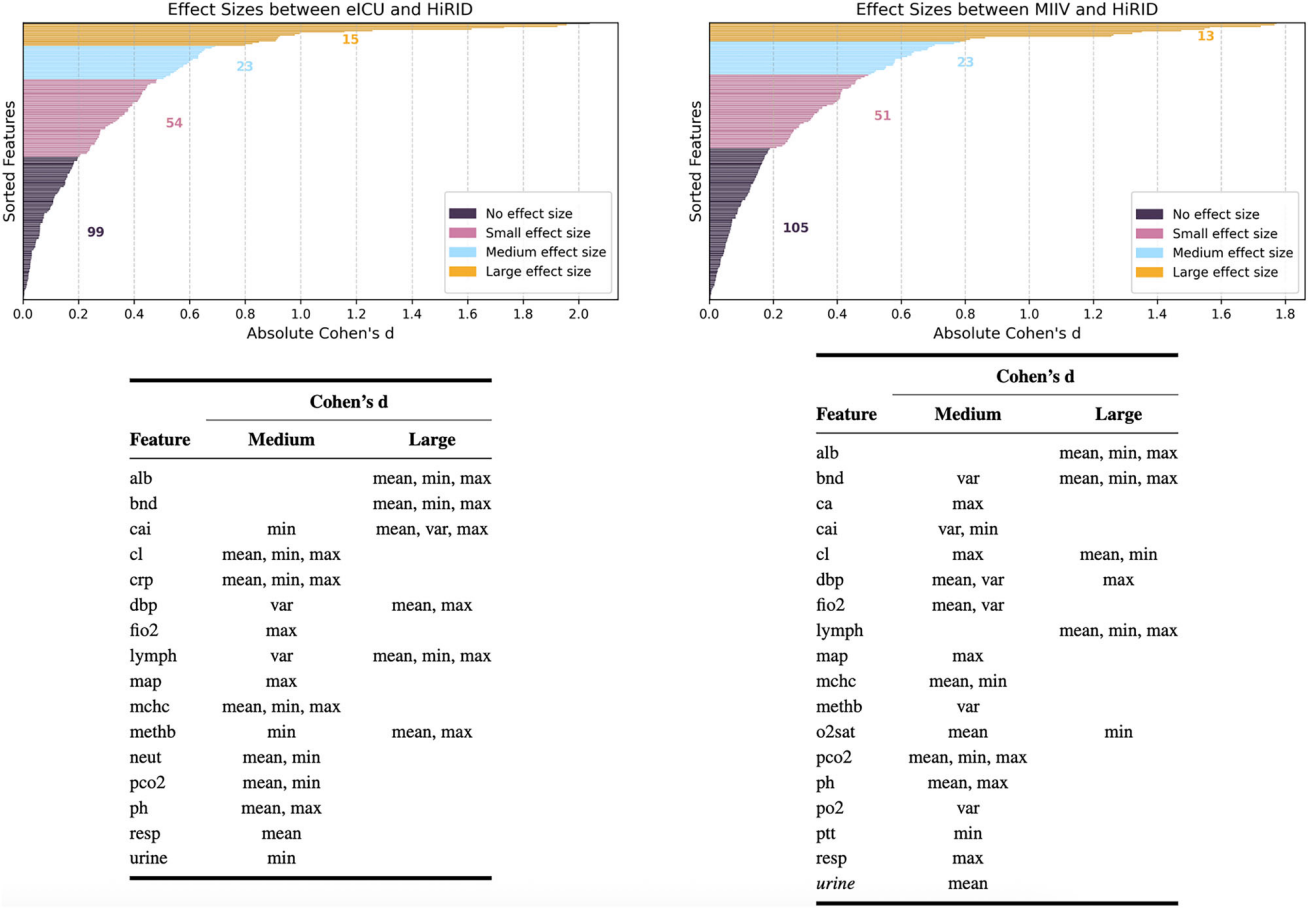
Representation of Cohen’s d analysis of Swiss-American dataset pairings (eICU-HiRID (left) and MIIV-HiRID (right)). Top row: Visualization of the number of features into effect size categories based on absolute Cohen’s d. Bottom row: Classification of features into medium and large effect size categories.

To confirm the findings, as another alternative method, Cohen’s d offered a detailed perspective on the extent of the shifts in the feature distribution among the three pairings of datasets. In particular, the pairing of the American dataset (MIIV-eICU) exhibited fewer descriptive statistical characteristics with small, medium, or large effect sizes compared to the two Swiss-American pairings (MIIV-HiRID, eICU-HiRID). Figures 2 and 3 illustrate these differences, highlighting that distribution shifts were generally more pronounced when the data were related to the Swiss ICU (HiRID). Within these Swiss-American comparisons, 18 and 16 dynamic features showed medium or large effect sizes for MIIV-HiRID and eICU-HiRID, respectively, whereas the MIIV-eICU pairing contained only seven dynamic features with such effect sizes. Specifically, five dynamic features (band form neutrophils (*bnd*), calcium ionized (*cai*), fraction of inspired oxygen (*fio2*), methemoglobin (*methb*), and urine output) had unique distributions in each dataset. Meanwhile, seven other features (albumin (*alb*), chloride (*cl*), diastolic blood pressure (*dbp*), lymphocytes (*lymph*), mean arterial pressure (*map*), mean corpuscular hemoglobin concentration (*mchc*), and respiratory rate (*resp*)) appeared specific to HiRID, in the sense that MIIV and eICU exhibited more similar distributions relative to each other. These observations suggest that datasets originating from the same country (e.g., MIIV and eICU in the United States) may have more comparable data characteristics than datasets from different countries (i.e., HiRID in Switzerland vs. the two American datasets). To examine the intricacies of these shifts, in Supplementary Section G, we further investigated distribution patterns for several dynamic features, visualizing them through violin plots and stratifying by the outcome of sepsis. These findings also show that feature-wise distribution shifts can be large and highly variable in different ICUs, further posing questions of whether naively pooling data (fusion training) or deploying models without adaptation (generalization) may fail to capture subtle, but critical, differences between sites in clinical practice and measurement protocols. Another argument could be that such a pooling of data could lead to more robust models, which is also to be examined in the next sections.

### Generalizability of deep learning models across ICU datasets

Tables 1 and 2 present the results for the generalization of the model and training of the target in three deep learning architectures: convolutional neural networks (CNN)=, InceptionTime, and long-short-term memory networks (LSTM). The column labeled “Generalization” denotes model performance when trained on a source domain and directly evaluated on a target domain without additional training. We also report the performance of the model on the target test set when trained with varying proportions (5%, 10%, 20%, 50%, 75%, and 100%) of the target domain training data.

**Table 1:** AUROC performance of models is evaluated from the source to the target

			Target training					
Source to target	Model	Generalization	5%	10%	20%	50%	75%	100%
**MIIV to HiRID**	CNN	0.676	0.567	0.679	0.675	0.699	0.750	0.748
	InceptionTime	0.647	0.634	0.672	0.677	0.675	0.721	0.767
	LSTM	0.655	0.650	0.649	0.674	0.728	0.719	0.720
**eICU to HiRID**	CNN	0.689	0.567	0.679	0.675	0.699	0.750	0.748
	InceptionTime	0.661	0.634	0.672	0.677	0.675	0.721	0.767
	LSTM	0.647	0.650	0.649	0.674	0.728	0.719	0.720
**HiRID to MIIV**	CNN	0.665	0.523	0.686	0.718	0.751	0.736	0.750
	InceptionTime	0.627	0.495	0.649	0.697	0.758	0.758	0.755
	LSTM	0.642	0.589	0.687	0.719	0.735	0.748	0.736
**HiRID to eICU**	CNN	0.610	0.624	0.630	0.617	0.679	0.696	0.702
	InceptionTime	0.597	0.612	0.601	0.648	0.691	0.693	0.703
	LSTM	0.614	0.634	0.615	0.640	0.679	0.691	0.701

The deep learning model types include CNN, InceptionTime, and LSTM. Generalization refers to the performance of the source model on the target testing set. The percentages 5%, 10%, 20%, 50%, 75%, and 100% represent the performance achieved when that proportion of the training set is used to train a model (target training) and then evaluated on the target testing set. The pink color indicates that the target training performance is better than the generalization performance. The green color indicates that the target training performance is less than the generalization performance.

**Table 2:** Normalized AUPRC performance of models is evaluated from source to target

			Target Training					
Source to target	Model	Generalization	5%	10%	20%	50%	75%	100%
**MIIV to HiRID**	CNN	1.979	1.186	2.242	2.067	2.177	2.791	2.732
	InceptionTime	1.622	1.715	2.202	2.104	2.143	2.450	2.987
	LSTM	2.096	1.861	1.749	1.978	2.456	2.673	2.286
**eICU to HiRID**	CNN	1.944	1.186	2.242	2.067	2.177	2.791	2.732
	InceptionTime	1.850	1.715	2.202	2.104	2.143	2.450	2.987
	LSTM	1.702	1.861	1.749	1.978	2.456	2.673	2.286
**HiRID to MIIV**	CNN	1.823	1.204	2.107	2.130	2.376	2.622	2.672
	InceptionTime	1.573	1.041	1.770	1.875	2.714	2.424	2.540
	LSTM	1.664	1.289	2.270	2.000	2.270	2.473	2.479
**HiRID to eICU**	CNN	1.615	1.476	1.554	1.447	1.849	2.033	2.132
	InceptionTime	1.432	1.443	1.362	1.544	2.073	1.957	2.128
	LSTM	1.539	1.518	1.353	1.597	1.852	1.962	2.072

The deep learning model types include CNN, InceptionTime, and LSTM. Generalization refers to the performance of the source model on the target testing set. The percentages 5%, 10%, 20%, 50%, 75%, and 100% represent the performance achieved when that proportion of the training set is used to train a model (target training) and then evaluated on the target testing set. The pink color indicates that the target training performance is better than the generalization performance. The green color indicates that the target training performance is less than the generalization performance.

Overall, these results confirm that pre-trained models offer clear advantages primarily in scenarios with limited target-domain data. Specifically, with only 5% of the target data available, using pre-trained models consistently yielded better performance compared to training solely on the limited target dataset. Among the architectures evaluated, CNN models demonstrated slightly higher generalization capabilities, performing robustly across diverse datasets. Both AUROC and normalized area under the precision-recall curve (nAUPRC) steadily improved as more target-domain data became available, eventually reaching a plateau. Although MIIV and HiRID reached comparable performance levels in 100% data use, the eICU dataset consistently exhibited lower performance by at least 0.05 AUROC and nAUPRC, indicating structural or demographic differences inherent to the patient population of the eICU or data collection methods, thus limiting achievable performance.

Dataset-specific observations are provided below: *HiRID as target domain —* Models trained or adapted for HiRID exhibited significant performance variability across different training set sizes. With only 5% of target data available, pre-trained models notably outperformed models trained exclusively on the small subset, with AUROC improvements of up to 0.12 for CNN models (particularly when sourced from the eICU). Notably, models pre-trained on eICU data generalized better than those sourced from MIIV, indicating higher robustness from eICU-trained models. As the target subset expanded to 10% and 20%, the AUROC values for target training and generalization converged, although nAUPRC remained higher for models trained directly on HiRID data. Once 50% or more of HiRID data became available, (*in-domain* target training consistently outperformed generalization. Thus, in our experimental setting, pre-trained models are beneficial in highly data-limited settings (up to approximately 20% of the target data), but direct target training becomes beneficial once moderate amounts of HiRID data (over 50%) are available. *MIIV as target domain —* All three architectures showed consistent AUROC trends, although nAUPRC exhibited some variability. With limited target data (5%), pre-trained models outperformed direct training on MIIV alone. However, as target data availability increased beyond 10%, direct training in the larger MIIV subset exceeded the generalization of source-trained models. In particular, AUROC performance plateaued around the 50% data threshold, while nAUPRC continued to improve slightly with additional data. This discrepancy suggests that the AUROC stabilizes earlier, whereas precision-recall metrics continue to improve incrementally with additional data, possibly highlighting the importance of data volume for precision-recall outcomes in our experimental settings. *eICU as target domain —* On the contrary, eICU exhibited relatively stable performance curves with minimal variability across different subset sizes. At the smallest training fraction (5%), direct training provided marginal AUROC improvements over generalization from pre-trained models. As training subsets expanded, performance plateaued markedly around two levels: an initial plateau at 5 to 20% and a second at 50 to 100% of available data. The narrower gap between these plateaus in the eICU compared to HiRID and MIIV may be explained by the greater inherent dataset diversity (reflecting its multi-center nature) or structural characteristics that make it easier to robustly model the prediction of sepsis, even with limited samples.

These results suggest that models pre-trained on source domains offer advantages in target scenarios with limited data, though these benefits decrease as more target-domain data becomes available. Furthermore, variations in clinical practices and demographics, particularly as observed in the eICU, indicate the relevance of dataset-specific factors when using external datasets for sepsis prediction.

### Systematic Comparison of the Five Deployment Strategies

**Fig. 4:**
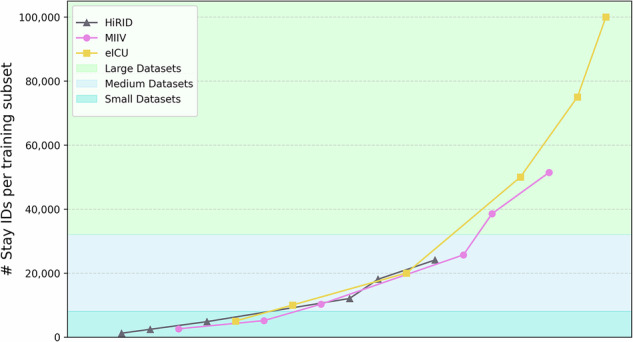
Visualization of dataset size groupings across HiRID, MIIV, and eICU training subsets. The figure illustrates the 18 training subsets (six subsets for each dataset: HiRID, MIIV, and eICU) categorized into small, medium, and large size groups based on the number of stay_ids. The subsets are arranged in ascending order of size, with each point representing a specific subset percentage (5%, 10%, 20%, 50%, 75%, and 100%) within its respective dataset.

In Section 2.2, we discussed how generalization and target training compare to each other. Next, we systematically evaluated the other four approaches to training or adapting deep learning models: fine-tuning, retraining, supervised domain adaptation (DA), and fusion training, across four source-to-target dataset combinations (Table 5). We also compare these performance against generalization (baseline) and target training performance, discussed earlier. The goal is to identify how effectively each approach leverages target data under varying sizes and conditions, since we already established that these datasets have considerable distribution shifts. Figure 6 shows the AUROC and nAUPRC box plots, illustrating the performance of each method by the combination of source-to-target and the size of the target dataset (small, medium, large, as described in Fig. 4). In all these figures, darker tone colors () represent DA methods, mid tone colors () indicate standard transfer learning approaches, and lighter tone colors () indicate fusion training. Moreover, each source-to-target combination is assigned a distinct color. For example, MIIV to HiRID in , HiRID to MIIV in , eICU to HiRID in , and HiRID to eICU in .

**Fig. 5:**
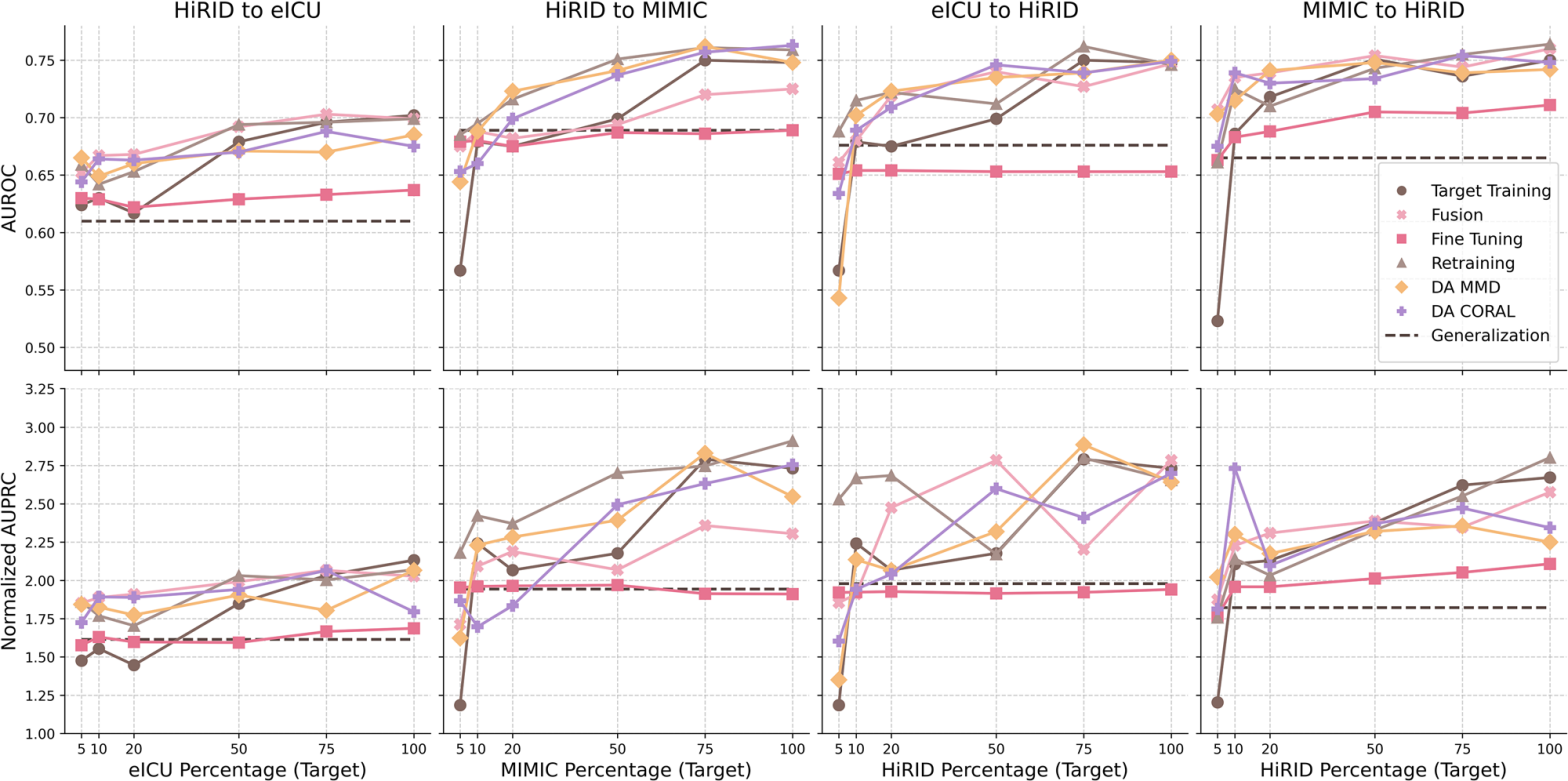
Comparison of CNN model performance across different strategies, for different source-to-target pairs. Each subplot shows the AUROC (top row) and normalized AUPRC (bottom row) as a function of the target dataset percentage used in the training process. The dashed brown line represents the Generalization baseline (performance of the source model in the target domain). Colored solid lines correspond to different training strategies, including Target Training, Fusion, Fine Tuning, Retraining, DA MMD, and DA CORAL.

First, if we consider the scenario where only a pre-trained model from the source domain is available, along with incrementally increasing amounts of target domain data (small [S], medium [M], and large [L]), the feasible strategies include generalization, fine-tuning, retraining, and direct target training. The results comparing these approaches across four distinct combinations of source-to-target datasets, using a CNN model, are shown in Fig. 5. As established in “Generalizability of Deep Learning Models Across ICU Datasets”, except for scenarios involving minimal target training data (approximately 5–10%), direct target training generally outperforms the straightforward generalization approach. This trend is clearly evident in the results presented. Comparing standard transfer learning methods (fine-tuning or retraining) with direct target training reveals that CNN-based models that utilize retraining consistently outperform direct target training in the overwhelming majority of cases. Similar patterns are observed for InceptionTime and LSTM architectures, although to a lesser extent. This finding suggests that, irrespective of the quantity of available target domain data, retraining a pre-trained source model typically yields superior model performance compared to training from scratch on the target domain alone.

**Fig. 6:**
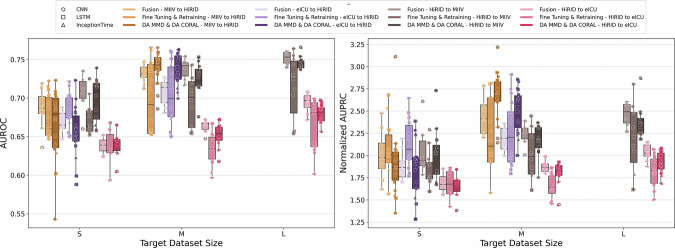
Joint DL models’ performance grouped by source-to-target combination, target dataset size (small, medium, and large), and transfer learning techniques.

Next, if we consider the scenario in which both the data and the model from the source domain are available alongside incremental target domain data, all six strategies become viable. In a more constrained scenario, where only the source data (but not a pre-trained model) are accessible along with the target data, the options reduce to direct target training, fusion training, and supervised domain adaptation. The results of these setups are presented in Fig. 6 as boxplots and are detailed in Figure 5. The boxplot results for both AUROC and nAUPRC indicate that, for medium-scale target datasets, supervised domain adaptation techniques clearly outperform more simplistic methods, such as fusion training or standard transfer learning. Even for smaller target datasets, supervised domain adaptation yields the highest peak performance across all models, particularly evident for CNN architectures, as illustrated in Supplementary Section C. Moreover, regardless of the size of the source dataset, domain adaptation consistently exhibits lower performance variability (illustrated by shorter boxes in the plots), indicating a more stable and robust generalization. In small data contexts, supervised domain adaptation outperforms standard transfer learning predominantly when the source domain dataset is sufficiently large (such as MIIV or eICU). In contrast, standard transfer learning approaches demonstrate greater variability (longer boxes), underscoring their sensitivity to differences in dataset size and composition.

Further analysis based on nAUPRC emphasizes a clear advantage when leveraging larger or medium-sized source datasets, such as eICU (represented in purple) or MIIV (in orange). Models trained on these datasets generally outperform those initialized from smaller source datasets (shown in gray and pink). Furthermore, the characteristics of specific datasets have a considerable impact on model performance. For example, models trained on the HiRID source dataset achieve better results when applied to the MIIV target dataset compared to the eICU target dataset, regardless of target subset size. This observation implies that the compatibility and similarity of the dataset, beyond the mere quantity of data, largely affect the performance. Furthermore, comparisons among the three deep learning (DL) architectures highlight the interaction between model design and dataset size. As illustrated in Supplementary Section C, the CNN and LSTM architectures generally follow the observed trends, with CNN showing slightly lower variance and consistently outperforming LSTM, demonstrating robustness across various domain changes. InceptionTime exhibits the greatest performance variability but performs notably well for small target datasets, particularly when paired with larger source datasets (MIIV or eICU).

**Fig. 7:**
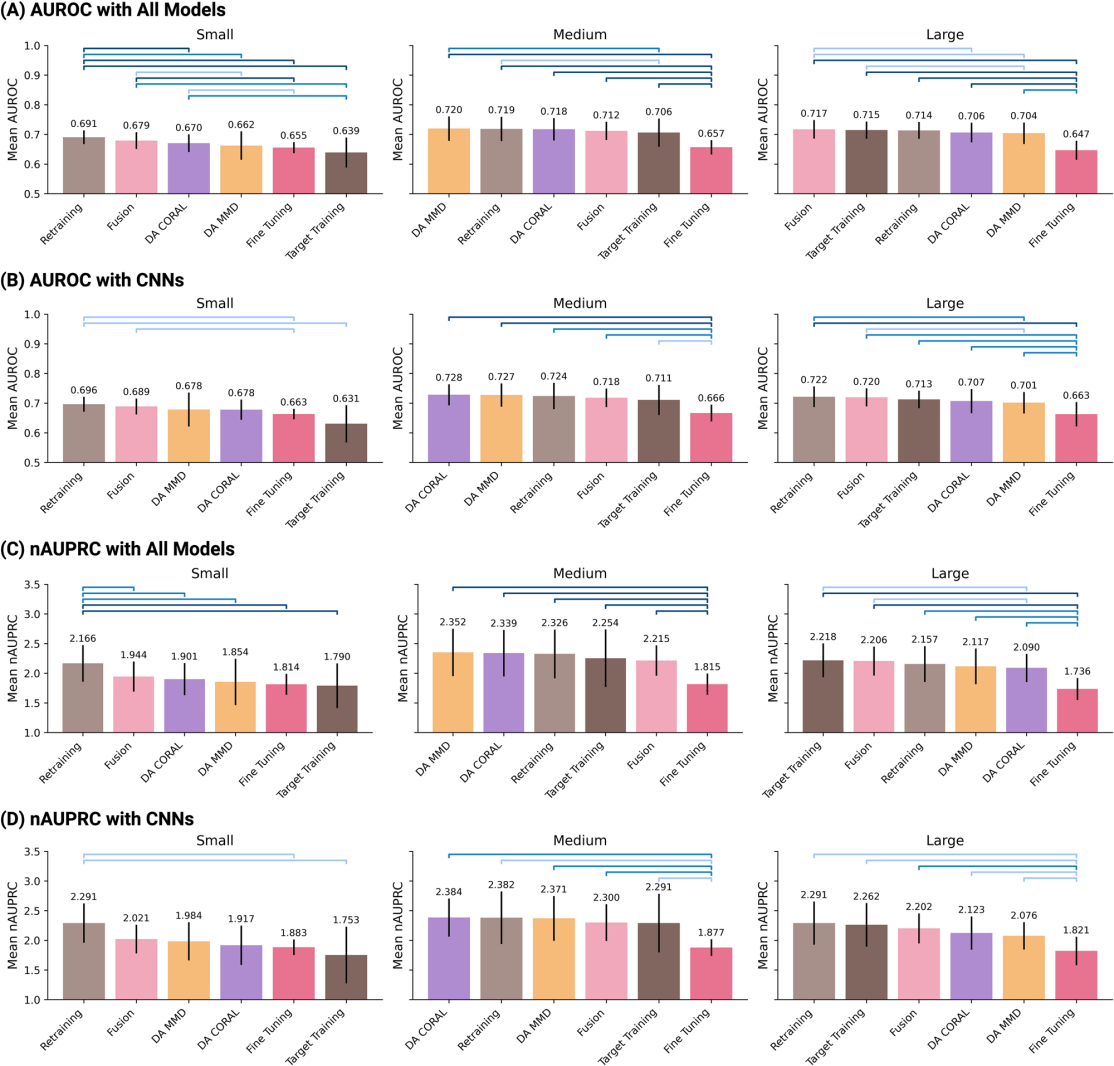
Rankings of strategies for models based on AUROC and nAUPRC. Colored brackets indicate statistically significant differences between pairs of methods: adjusted *p*-value < 0.05: ; adjusted p-value < 0.01: ; adjusted p-value < 0.001: . **A** AUROC for all models combined; **B** AUROC for CNNs only, the generally top-performing model; **C** nAUPRC for all models combined; **D** nAUPRC for CNNs only, the generally top-performing model.

To summarize all these results across different sources-target domain pairings, we created a ranking as given in Fig. 7, where for each target domain, dataset size (small, medium, and large), the performance of the approaches is averaged and ranked from highest to lowest, while also showing differences between different approaches using colored brackets. The results here confirm and synthesize our findings that for all models (Figure 7A, Figure 7C) and even for CNNs specifically (Figure 7B and Figure 7D), re-training is the superior method when the target dataset size is small, followed by fusion training. For medium-sized target domains, DA MMD and DA CORAL were superior. For large target domains, CNN models worked the best with retraining, whereas the highest performance when all models were combined was achieved with Fusion training (in terms of AUROC) and target training (in terms of nAUPRC). Furthermore, as the brackets indicating statistical significance show, highest performing techniques (retraining, DA, fusion) showed statistically significant performance improvements over commonly explored approaches such as fine-tuning and target training, particularly for small and medium target domains.

In summary, the findings indicate that supervised domain adaptation (e.g., MMD or CORAL) can be an effective strategy for medium-sized target datasets, often outperforming fusion training, generalization, or direct target training. The size and compatibility of the source dataset appear to influence outcomes, with larger sources (e.g., eICU to HiRID) generally associated with improved performance. Overall, the analysis suggests that aligning training and deployment strategies, including the choice of transfer learning methods and architectures, with the specific characteristics of the data may improve the predictive performance of sepsis models. While fine-tuning is a common standard in the literature, these results imply it may not be the optimal approach in every scenario.

## Discussion

Our findings have implications for the deployment of AI sepsis prediction models in ICU settings. The results indicate that distribution shifts between hospitals are a practical factor that can influence model performance. Consequently, a model developed at a large center may perform differently on a new patient population without adjustment. This highlights the value of local validation prior to clinical use, as performance metrics may vary due to the shifts quantified in this study. However, the analysis also demonstrates strategies that can help mitigate these challenges.

Domain adaptation provides a tangible approach to improve the generalizability of the model across different ICUs, even with a small amount of labeled target data. In practical terms, a hospital adopting an externally developed sepsis model can apply domain adaptation by using a modest set of its own data (perhaps a few months of retrospective data) to fine-tune the model in a domain-aware way. For example, suppose that Hospital B wants to use a hospital-acute sepsis model. Rather than training a new model from scratch (which could take years of data collection), Hospital B could use Hospital A’s data as well and train on a sample of 1000 cases of sepsis and non-sepsis from Hospital B, while using a DA technique to ensure that the model’s internal representations adjust to fit Hospital B’s data patterns. Our results suggest that this process can produce a model nearly as good as if Hospital B had years of its own data to train with. This has huge time and cost benefits; it accelerates the availability of AI tools in settings that lack big data resources. Furthermore, because the domain adaptation uses source data during training, the adapted model in Hospital B still benefits from the larger dataset of Hospital A (for general sepsis aspects), but also fits the specific profile of Hospital B (for differences, such as laboratory value distributions or patient demographics).

From a clinical perspective, the ultimate measure of success is whether these models actually improve patient outcomes by requiring earlier or more accurate interventions. A model that performs poorly in a new environment risks generating false alarms or missed detections, undermining clinician trust and potentially compromising patient safety. By mitigating performance degradation, domain adaptation can ensure that predictive accuracy remains high in the new setting, preserving the positive impact on patient care. For example, if without adaptation the model sensitivity to sepsis in Hospital B had dropped, clinicians might miss the early window to treat sepsis in some patients; with adaptation that restores sensitivity, these cases can be caught. Likewise, specificity might be maintained, avoiding alarm fatigue from too many false alerts in the new unit. Basically, DA contributes to safer and more reliable AI. We also found that certain model architectures (such as CNNs) are inherently more stable across domains, suggesting that choosing the right model design is part of making an AI tool robust for deployment. Simpler models that generalize well may be preferable to complex ones that achieve slightly higher accuracy in one setting but fail to transfer. Clinicians and engineers who collaborate on AI should consider this trade-off: a model that is 2% less accurate in one hospital but portable in many hospitals may have a greater overall impact on patient outcomes than a model that is highly optimized in one place only.

Another practical insight is our guideline on when to update a model with local data and when it might be unnecessary. For example, if a hospital is implementing an ICU sepsis model and has virtually no local training data, our results suggest using the model as-is might yield suboptimal performance, but collecting even a small amount of data (a few hundred cases) to adapt the model will produce large gains. On the other hand, if a hospital has a very large trove of its own ICU data (tens of thousands of cases with results), it might consider training its own model or doing extensive retraining, as the marginal benefit from external data is small. Many real-world scenarios will fall between moderate data availability. For those, our work indicates that joint training with domain adaptation is likely the best route, rather than pure fine-tuning. Hospital systems that have multiple sites could use a domain adaptation strategy to create a network of models: a central model plus adapted versions for each site, sharing knowledge but tuned to local peculiarities. Moreover, we would also like to emphasize that, while our experiments center on ICUs, the data harmonization and transfer/adaptation insights are portable to ED and ward settings^24^ where sepsis-related inferences could be useful. Future work could examine whether the strategy ranking we report (retraining for small data, DA for medium, fusion/target for large) holds outside the ICU.

We also highlight that simply merging data from different sites into one large training set is not necessarily a panacea. Although having more data is generally beneficial, our analysis of distribution shifts shows that without explicitly handling those shifts, a merged model may still underperform in each constituent domain. For example, it could have a calibration problem: perhaps systematically overpredict risk in one hospital and underpredict in another if data from one site dominates training. Clinically, this could mean that one hospital’s clinicians receive too many alerts and another too few. A smarter approach might be to merge the data but include domain adaptation or stratification in the modeling process (e.g., domain-specific layers or parameters for each hospital). Our results on the benefits of DA hint that such an approach would outperform a naive pooled model. This is an area for future exploration (and in fact, we suggested ’fusion training’ with careful consideration as future work).

Lastly, from a clinical governance standpoint, our study underscores the importance of data standards and sharing. The fact that we had to harmonize three datasets and still found so many differences highlights how variable healthcare data can be. If in the future more hospitals adopt common data standards (for example, using common unit measurement and documentation practices), distribution changes might be lessened, making generalization easier. However, given the reality of the differences between patients and practice, some level of change will always persist. Therefore, having methods like domain adaptation in the toolbox can facilitate the translation of medical AI innovations from one setting to another. This is analogous to how a drug developed in one population might need dose adjustments in another; an AI model might need a ’dose adjustment’ in the form of retraining or adaptation when moving to a new ICU population.

Although this study provides important information, it also has several limitations that point to areas for further caution and research. First, our work is retrospective in nature. We evaluated model performance using historical ICU data from three databases and, while we simulated the effect of deploying a model in a new domain, we did not prospectively implement these models in real ICU workflows. Thus, factors such as the availability of real-time data, integration with clinical systems, and human-in-the-loop considerations were not tested. Some practical issues (e.g., missing data that arrive late or clinicians reacting to model outputs) could affect the performance of a model deployed in ways not captured here. For example, the exact timing of sepsis onset may be difficult to identify with complete certainty using datasets such as these. Missing or retroactively filled-in data may fail to capture all the necessary details of patient development. Missing data specifically is a challenge for sepsis prediction^25,26^, where high-resolution data relies on careful imputation. For this work, we focused primarily on data scarcity rather than missingness, and employed the widely accepted standard imputation methods used in the literature^1,11,18^. However, it is important to note that models can be quite sensitive to imputation methods^25,27,28^, and further research that focuses on data missingness directly would also be very useful. Taking this idea further, future work should investigate ICU datasets that are inherently more limited than the publicly available datasets used here. The three datasets in this study are from the United States and Switzerland, countries with highly developed healthcare systems. More generally, publicly available ICU datasets tend to originate from the US, Europe, or China^29^. However, many countries around the world have ICUs that lack the resources needed to generate such rich, high-resolution time-series data^30,31^. Addressing the needs of these resource-limited patient populations is critical and motivates research into model development using fewer or more basic clinical features. Prior work has already shown that models trained on richer data can struggle to generalize to more restrictive settings^32^. In addition, future datasets could capture more nuanced contextual information beyond time-series clinical measurements, such as resource constraints, staffing levels, and care pathways, to better characterize the environments in which these models are intended to operate. Moreover, some retrospective datasets (e.g., HiRID) may lack richer clinical narrative/context beyond physiologic streams. We therefore focused on variables required for Sepsis-3 labeling and harmonized time-series features; future work should incorporate clinician-adjudicated context or free-text to mitigate residual confounding. Along the same topic, this study emphasizes threshold-independent metrics (AUROC and nAUPRC) to enable cross-dataset comparison under class imbalance and shift. We did not compute task-specific utility or cost-sensitive metrics because the necessary intervention, workflow, and cost annotations are not available in MIMIC-IV, eICU, or HiRID. Such utility formulations are setting-dependent and are best assessed prospectively alongside alarm burden and treatment timeliness. Future work should pair model outputs with workflow logs and cost assumptions to quantify net benefit in deployment, and examine how such models perform under distribution shift.

Second, we examine only a subset of possible domain-adaptive techniques. We chose MMD and CORAL for their simplicity and proven performance in other domains, but there are more advanced methods. For example, adversarial domain adaptation (such as training a model to fool a domain discriminator, as done in Domain-Adversarial Neural Networks)^33^ could potentially learn even more invariant features. Generative approaches or normalizing flows could explicitly transform data from one domain to another distribution. Furthermore, recent techniques in transfer learning, such as fine-tuning with discriminative learning rates or parameter-efficient transfer (like freezing most of the model and only learning small adapter modules), were not explored and could be beneficial in this context. This work also focuses on a single high-level adaptation strategy applied equally to all data features, rather than a feature-specific approach. However, building upon the analysis in Figs. 2 and 3, one promising approach would be to adapt each feature separately, using its own parameter/weight for adaptation strength. This would allow the model to adapt more explicitly to the underlying differences between the source and target sets, at a high level of granularity. Our results open the door to testing these methods: given that we have established sepsis prediction as a problem domain where adaptation helps.

Third, our analysis of distribution shifts was somewhat high-level. We identified and quantified differences in feature distributions, but we did not delve deeply into the root causes of each difference, and also the clinical implications of these findings. For example, we note that the neutrophil values of the band ranged from 0 to 100% in one dataset versus 0 to 10% in others; this could be due to different laboratory reports (perhaps one reports band forms as a percentage of WBC, another as an absolute count, or different lab standards). We did not attempt to standardize such features beyond what we did initially; perhaps some differences could be eliminated by better data pre-processing (unit conversion, outlier handling) if one knows the cause. We also did not explore concept drift or label differences, and we assumed that the Sepsis-3 criteria were applied uniformly, but it is possible that the way sepsis onset is identified in each dataset had subtle differences (e.g., exactly how infection suspicion was determined from the chart data). If the sepsis labels themselves are slightly inconsistent, that could introduce another kind of shift (target shift or label shift), which we did not explicitly address. Our focus was on covariate shift (features), and we treated the prediction task as identical across domains. In reality, one limitation is that sepsis detection criteria might vary or have noise; something that future work could investigate by, say, using an alternate sepsis definition or physician adjudication to see if results hold.

Additionally, this study was limited to three specific datasets (MIMIC-IV, eICU, HiRID). All are large, high-quality clinical research databases from tertiary ICUs in the United States and Switzerland. They may not capture the full spectrum of ICU types (for example, community hospital ICUs, pediatric ICUs), and conditions differ substantially from those in many resource-limited settings, where measurements may be sparse or unavailable and where our conclusions may not directly apply. Thus, the shifts we observed (geographic and practice-related differences) might differ in other settings (for example, shifts driven by temporal changes or by the use of a different EHR system). While we expect the general principles to hold, the specific feature distributions and the magnitude of the observed effects may vary. We therefore encourage validation of our findings on additional datasets as they become available (for example, emerging multi-center ICU datasets from other countries). Furthermore, we only examined adult sepsis; pediatric sepsis prediction is another area where data are more scarce, and inter-hospital differences (e.g., between children’s hospitals) may be substantial, potentially making adaptation even more critical. Finally, we did not evaluate transfers between MIMIC-IV and eICU because possible cross-hospital overlap within the United States cannot be audited with the currently available de-identified metadata, and we found no authoritative source guaranteeing non-overlap. To minimize the risk of data leakage, we focused on transfer directions that are verifiably disjoint by geography or curation. If future releases include auditable site information, these directions could be revisited with appropriate safeguards.

## Methods

### Dataset harmonization and pre-processing

We used three open, high-resolution ICU databases to ensure a diverse representation of patient populations and clinical practices. MIMIC-IV (a single-center US ICU database)^34^, eICU Collaborative Research Database (a multi-center US ICU database)^35^, and HiRID (a single-center Swiss ICU database)^36^. Collectively, these databases encompass more than 216,000 stays in the ICU, including more than 10,800 patients who developed sepsis during their ICU course. All three datasets were filtered to include adult stays in the ICU and annotated with sepsis outcomes based on Sepsis-3 criteria (which require suspicion of infection plus organ dysfunction). We harmonized variable definitions and units across databases using a rigorous pre-processing pipeline. This involved mapping the raw features of each dataset to a common set of clinical variables (e.g., vital signs such as heart rate, lab measurements such as creatinine) with consistent units and naming. We focus on 48 time-varying physiological features available in all three sources, along with relevant demographics. Continuous signals were resampled or summarized into hourly intervals to allow alignment. Outliers were handled by capping physiologically implausible values, and each time series was filled forward and imputed as needed to handle missingness. To ensure label consistency, we applied the same sepsis onset detection logic (based on the increase in the Sequential Organ Failure Assessment score and the clinical documentation of infection) uniformly to all datasets, verifying that the incidence rates matched published values for each database.

For dataset harmonization, we closely followed the methodology outlined in YAIB^18^. By adopting this framework, we ensured consistency and reproducibility in the dataset harmonization process, without having to make individual decisions regarding data preprocessing, cohort definitions, or task evaluation. Hence, the HiRID, MIIV, and eICU datasets were harmonized, ensuring that all datasets shared the same set of characteristics and structure. Specifically, the harmonized datasets included four static features and 48 dynamic features (7 vitals, 39 lab tests, urine output, and fraction of inspired oxygen). These features were available on an hourly basis from day 1 to day 7 of the ICU stay. To define sepsis, we adhered strictly to the Sepsis-3 criteria, including the length of antibiotic treatment and body fluid culture to characterize suspicion of infection. In addition, to confirm sepsis, antibiotics were required to be administered continuously for at least three days. For labeling purposes, the sepsis label was set to “True”, six hours before the confirmed onset of sepsis. This allowed the model to account for early warning signs of sepsis, hence making the task we attempted a sepsis prediction task six hours in advance. Regarding the temporal aspect, a maximum of 13 hours with the “True” label was kept after the onset of sepsis, to make sure that too distant data points from the onset are considered, making the inference easier and biased. We then excluded patients who had sepsis prior to ICU admission, as well as those who developed sepsis within 6 hours after admission. This ensured that training samples always contained sufficiently long prediction windows of data from the ICU. The total number of stay_ids present in each of the datasets after harmonization as well as the total number of stay_ids that developed sepsis, are presented in Table 3.

**Table 3:** Number of Stay_ids within the harmonized datasets

Dataset	eICU	MIIV	HiRID
Total number of stay_ids	123,413	63,425	29,698
Total number of stay_ids with sepsis	5,638	3,321	1,858
% of stay_ids with sepsis	4.568%	5.236%	6.256%

To further preprocess the harmonized ICU datasets, several steps were taken to ensure that the data were clean, standardized, and suitable for training DL models. Each dataset was divided into three subsets according to the timestamp: 80% for training, 10% for testing, and 10% for validation. This division ensured that the model had ample data for training while also having separate data to evaluate its performance during training (validation) and after training (testing). Importantly, all data were stratified by stay_id and label, preventing any data leakage and ensuring that each split contained a balanced representation of patient stays across all three subsets. The number of stay_ids present in each of the three splits is displayed in Table 4.

**Table 4:** Training, testing and validation splits

Dataset		eICU	MIIV	HiRID
**Training**	Total number of stay_ids without sepsis	95,358	48,640	22,528
	Total number of stay_ids with sepsis	4605	2733	1527
	Total number of stay_ids	99,963	51,373	24,055
**Testing**	Total number of stay_ids without sepsis	11,787	6046	2809
	Total number of stay_ids with sepsis	555	297	170
	Total number of stay_ids	12,342	6343	2979
**Validation**	Total number of stay_ids without sepsis	10,630	5418	2512
	Total number of stay_ids with sepsis	478	291	161
	Total number of stay_ids	11,108	5709	2673

Given the high frequency of missing values, particularly in dynamic features (i.e., lab results), some strategies were used to handle missing values. Initially, missing values in dynamic features were flagged, creating an additional binary column for each feature that indicated whether a value was missing. This resulted in every dynamic feature having two corresponding columns: the original value and the missingness flag. After flagging, the missing values were re-filled, which means that they were replaced with the last available measurement for the same patient during their ICU stay. If no prior measurement was available for a feature, the missing value was filled with the mean of that feature, calculated solely from the training set to avoid data leakage. This means imputation was determined before any forward filling was applied, ensuring a consistent and unbiased approach throughout the dataset. To prepare the dynamic features for model training, they were standardized using the StandardScaler function from the Scikit-learn library. This process scaled each feature to have a zero mean and unit variance. By standardizing the features, all variables were brought onto a comparable scale, ensuring that no single feature disproportionately influenced model training. Importantly, the scaling parameters (mean and variance) were computed using only the training set and then applied to both the validation and testing sets.

For model experimentation, the training set was further split into six progressively larger subsets: 5%, 10%, 20%, 50%, 75%, and 100%. In constructing these subsets, additional stay_ids were added sequentially to each larger subset. For example, the 10% subset included all stay_ids from the 5% subset plus 5% new stay_ids. Similarly, the 20% subset contained all stay_ids from the 10% subset, with an additional 10% of new stay_ids, and so on. This incremental addition ensured that each larger subset was an expansion of the previous one, preserving continuity in the dataset composition while enabling an evaluation of the impact of data size on performance. Importantly, each subset was stratified by stay_id and label to maintain a balanced representation of ICU stays across all training subsets. Evaluating different dataset sizes is particularly important given that this is the sequential nature in which data are collected and stored in ICUs, in the real world. Moreover, to capture the temporal dynamics of patient data, the data was segmented into 6-hour windows based on the stay_id of each patient. After extraction, the time and stay_id columns were removed from the datasets.

### Training deep learning models

To evaluate the performance of DL models on sepsis ICU datasets, we implemented three model architectures (see Supplementary Section A): Convolutional Neural Networks (CNN), InceptionTime, and Long-Short-Term Memory (LSTM) networks. These model architectures were chosen because they have shown decent performance for time series modeling tasks, including predictive tasks in the ICU. Model performance was assessed primarily using the Area Under the Receiver Operating Characteristic Curve (AUROC) and the normalized Area Under the Precision-Recall Curve (nAUPRC). We selected these metrics for three reasons. First, both AUROC and AUPRC are threshold-independent summary measures that characterize discrimination across all possible decision thresholds, which is particularly useful when comparing many models and training strategies without committing to a specific operating point. AUROC captures the sensitivity-specificity trade-off, while AUPRC summarizes the precision-recall trade-off, which is especially informative under class imbalance. Second, the sepsis label is relatively rare in some of our settings, and raw AUPRC is sensitive to the underlying prevalence. To account for this, we report a normalized AUPRC, defined as ${\rm{nAUPRC}}=\frac{{\rm{AUPRC}}}{{\rm{Baseline}}},\,{\rm{where}}\,{\rm{Baseline}}=\frac{{\rm{Positiveclasssamples}}}{{\rm{Totalsamples}}}.$ This normalization adjusts for differences in event rate and facilitates fairer comparisons across datasets and experimental conditions with varying class imbalance. Under this definition, an nAUPRC of 1 corresponds to baseline (random guessing) performance, and values greater than 1 indicate performance better than baseline. Third, AUROC and AUPRC are standard in the literature on critical care time-series and sepsis prediction, which supports comparability with prior work^11,20–23^. A threshold-based metric, Recall, is additionally reported in Supplementary Section D. Because missing sepsis cases are clinically costly, Recall directly quantifies each model’s ability to identify true sepsis episodes, complementing the threshold-independent AUROC and nAUPRC.

Models were trained using a batch size of 32, binary cross-entropy with logit loss (BCEWithLogitsLoss) as the loss function, with positive samples weighted to address class imbalance. Training employed early stopping, terminating if the validation loss kept increasing for five consecutive epochs. The best performing models, determined by the lowest validation loss, were saved. All architectures used the Leaky ReLU activation function to mitigate vanishing gradient problems for negative activations. Additionally, the same random seed was set across all models to ensure reproducibility. For the CNN model, we used the stochastic gradient descent (SGD) optimizer with a learning rate of 0.01, a momentum factor of 0.85, and a weight decay of 0.0001. A learning rate scheduler reduced the learning rate by a factor of 0.9 if the validation loss plateaued for five consecutive epochs, with a minimum learning rate of 0.001. Training was carried out for up to 150 epochs. The LSTM model was optimized using Weighted Adam (AdamW), with an initial learning rate of 0.001. A learning rate scheduler decreased the learning rate by a factor of 0.85 if the validation loss failed to improve for five consecutive epochs, with a minimum learning rate of 0.0001. The training process spanned 120 epochs. Lastly, for the InceptionTime model, a depth of 12 modules was used to extract hierarchical temporal features^37^. For this, we used the InceptionTime model architecture by Fawaz et al.^38^. The optimizer was AdamW with a learning rate of 0.01. A learning rate scheduler adjusted the learning rate by a factor of 0.9 based on stagnation of the validation loss, with a minimum value of 0.001. Training was limited to 60 epochs due to the model’s higher computational costs. Again, with early stopping, none of the models reached the maximum number of epochs we set when starting the model training.

### Existence and significance of distribution shifts between sites

First, to explore the presence and significance of covariate distribution shifts between ICU datasets, we performed a comparative analysis of the three datasets. Since the MIIV and eICU datasets originate in the United States, while the HiRID dataset comes from Switzerland, we also examined whether these shifts were more pronounced when comparing the datasets from the two countries. The analysis focused on the three dataset pairings: HiRID-MIIV, HiRID-eICU, and MIIV-eICU. The datasets analyzed were the unprocessed harmonized versions, which means that they retained missing values and had not undergone preprocessing steps such as forward filling or imputation (see “Dataset Harmonization and Pre-Processing”). This approach allowed for a more accurate assessment of the raw distributional differences between datasets. To quantify distribution shifts, we performed a statistical analysis. For each dynamic feature and stay_id, we calculated descriptive statistics including the mean, variance, minimum, and maximum values, resulting in a total of 192 features (48*dynamicfeatures* × 4*descriptivestatistics*). To assess the statistical significance of the shifts, we performed pairwise comparisons of the three dataset pairings using the two-sample Kolmogorov-Smirnov (K-S) test, with a Benjamini/Hochberg correction to account for multiple testing. Features with p-values less than 0.01 were considered significant. In addition to the statistical analysis, the importance of the distribution shifts was quantified by measuring effect sizes using the absolute Cohen’s-d. The shifts were classified as small effect size (0.2–0.5), medium effect size (0.5–0.8), or large effect size (greater than 0.8). This dual approach, including statistical testing and effect size estimation, aimed to provide a comprehensive view of the magnitude and relevance of the observed shifts, particularly in the context of datasets from different countries. To visually explore feature distributions, we created violin plots of the dynamic features of each dataset. For added granularity, we stratified each dataset based on patient stay_ids into two subgroups: (1) stays where patients developed sepsis, with distributions plotted separately for the periods before and during sepsis, and (2) stays where patients did not develop sepsis, focusing solely on the negative label distribution.

### Generalizability of Deep Learning Models Across ICU Datasets

Next, the dataset combinations presented in Table 5 were used consistently in the next set of experiments. These combinations were designed to evaluate the generalizability of sepsis detection models across ICU datasets and assess the impact of training data availability on model performance. The first set of experiments examined whether models trained on a source domain could maintain robust performance when tested on an unseen target domain without further training. The three DL architectures, LSTM, CNN, and InceptionTime, were trained using 100% of the source training dataset and evaluated on the target testing dataset. This setup allowed for a direct assessment of model generalizability under distribution shifts. In the second step, the focus shifted to the influence of target domain training data on model performance. Models were trained exclusively using progressively larger subsets of the target training data, comprising 5%, 10%, 20%, 50%, 75%, and 100% of the target dataset. Performance was evaluated on both the target testing and validation sets. This analysis compared the effectiveness of pre-trained models (without additional training) against newly trained models across varying data availability. The goal was to determine whether a pre-trained model could outperform newly trained models, even when only limited target data were available.

**Table 5:** Source and target datasets combinations

**Source Domain**		**Target Domain**
HiRID		MIIV
HiRID		eICU
MIIV		HiRID
eICU		HiRID

### Systematic comparison of the five deployment strategies

Finally, we evaluate techniques to enhance model generalization in the target domain, comparing standard transfer learning approaches (fine-tuning and retraining) with supervised DA methods (MMD and CORAL) and fusion training. Each approach was assessed using target training datasets of varying sizes (5%, 10%, 20%, 50%, 75%, and 100%), while the source training dataset was maintained at 100%. The same four source-to-target dataset combinations (Table 5) were used as in RQ2. The selection of the model was based on the lowest validation loss, with evaluations performed consistently on the target testing dataset. First, standard transfer learning techniques - fine-tuning and retraining - were applied to adapt pre-trained models to the target domain. Fine-tuning involved updating only the final fully connected layers of the pre-trained models, keeping the earlier layers frozen to preserve general feature representations learned from the source domain. This approach helps maintain the model’s ability to extract generalizable features while adapting to the target domain and reduces the risk of overfitting to smaller target datasets^8^. Adjustments were made to the learning rate scheduler to accelerate convergence during training, with factors set to 0.75 for CNN and InceptionTime and 0.7 for LSTM. Apart from those, no other changes were made. In contrast, full retraining updated all weights of the pre-trained models, using them as initialization for training on the target domain. This approach allows the models to adapt more comprehensively to the target dataset while retaining useful representations from the source domain, based on the initialization. To prevent overfitting and ensure stable optimization, optimizer learning rates were reduced, and the learning rate scheduler’s factor was divided by 10 to maintain an optimal ratio with the optimizer. In addition, dropout rates were increased, and dropout layers were added to enhance regularization, to avoid overfitting. Specific modifications for each architecture included—CNN: The optimization learning rate was set at 0.001, the probability of dropout increased from 0.5 to 0.6, and an additional dropout layer was added; LSTM: The optimization learning rate was set at 0.0001, and the dropout rates were uniformly increased to 0.6; InceptionTime: The optimization learning rate was set to 0.0001, the probability of dropout increased from 0.3 to 0.7, and a dropout layer was added after each InceptionTime module.

DA, another form of the advanced transfer learning strategy, incorporated labeled source and target data to compute a domain alignment loss, which aims to align feature distributions between the two domains. To address differences in dataset sizes, the smaller dataset (source or target) was repeated to match the size of the larger dataset within each epoch. The features were extracted for alignment after the model embedding layers, before the task classifier^39^. The total loss function combined classification losses from the source and target domains with the domain alignment loss scaled by the adaptation weight *λ*, calculated as: *TotalLoss* = *SourceLoss* + *TargetLoss* + (*λ* × *DALoss*). MMD used a Gaussian RBF kernel to align the statistical means of the source and target feature distributions in a shared feature space. We based our code on the implementation of Lee et al.^40^. Key hyperparameters included a fixed bandwidth length (*fix*_*sigma* = 1.0), the Gaussian RBF Kernel’s bandwidth parameter (*kernel*_*mul* = 2.0), and the number of Gaussian kernels with different bandwidths (*kernel*_*num* = 5). For CNN, a *kernel*_*mul* = 5.0 was chosen because *λ*-tuning did not yield satisfactory results. The adaptation weights *λ* were tuned in different combinations of the source and target datasets, and the value that provided the best overall performance was selected. *λ* was set to 1 for CNN and LSTM, and 10 for InceptionTime. Moreover, CORAL achieved alignment by minimizing the distance between the covariance matrices of the source and target feature embeddings. The implementation of CORAL was adapted from Github of the Institute of Computational Perception^39^. Adaptation weights *λ* were adjusted for each architecture and set to 1,000 for CNN and 10,000 for LSTM and InceptionTime. No additional hyper-parameters were tuned.

With these experiments, we also wanted to understand the effect of the source and target domain’s training dataset size on the model performance. In the previous experiments, models were trained on datasets of varying sizes. When using 100% of the training data, the total number of stay_ids was approximately 24K for HiRID, 51K for MIIV, and 100K for eICU (see Fig. 4). To systematically analyze whether the size of both source and target datasets influences a model’s generalization ability, as well as whether specific transfer learning methods perform better under certain conditions, we categorized the datasets into small, medium, and large groups. The training sets for the source domain were classified as follows: HiRID as small, MIIV as medium, and eICU as large datasets. For the target domain training subsets, categorization was based on the number of stay_ids, with small datasets containing up to 8000 stay _ids, medium datasets containing between 8000 and 32,000 stay_ids, and large datasets containing more than 32,000 stay_ids. To make our findings actionable for deployment, we pre-specified target-data size bins by *absolute* stay counts at the target site. Using absolute counts lets readers map their own cohorts without prevalence assumptions. We report results for each bin. These thresholds are pragmatic, not universal, and may shift with local prevalence, labeling quality, feature availability, and case mix. Future work could derive data-adaptive breakpoints from learning curves, alert budgets, and annotation costs. Table 6 maps each training subset to its respective group, while Fig. 4 visually illustrates these groupings. In the figure, each dataset (HiRID, MIIV, and eICU) is represented by six points corresponding to the six training subsets (5%, 10%, 20%, 50%, 75%, and 100%). These 18 training subsets (3 datasets × 6 subsets) are arranged in ascending order of dataset size for clarity. This categorization allows for a systematic exploration of how dataset size impacts model performance and enables comparison across different training scenarios.

**Table 6:** Training subsets in each size group

Dataset	eICU	MIIV	HiRID
**Small: 0–8000 stay**_**ids**	5%	5%, 10%	5%, 10%, 20%
**Medium: 8000–32,000 stay**_**ids**	10%, 20%	20%, 50%	50%, 75%, 100%
**Large: ≥ 32,000 stay**_**ids**	50%, 75%, 100%	75%, 100%	-

For each target size bin (small, medium, large), we collected every AUROC and nAUPRC value produced in “Systematic Comparison of the Five Deployment Strategies”. This produced, per deployment strategy, a matched set of 4 source → target pairs × 3 architectures = 12 scores (for the panels ’all models’) or 4 × 1 = 4 scores when the analysis was restricted to CNNs. Within a bin, we averaged those scores and assigned ordinal ranks (1 = best, 6 = worst; ties were broken by retaining additional significant digits). To test whether the leading strategy was meaningfully better than its competitors, we applied a paired Wilcoxon signed rank test to the matched score vectors of every pair of strategies, correcting *p*-values for multiple comparisons with the Bonferroni correction. The resulting significance levels are depicted in Fig. 7 as colored brackets (*p* < 0.05, *p* < 0.01, *p* < 0.001). This procedure was repeated independently for AUROC and nAUPRC, producing the four ranked panels: (A) all models/AUROC, (B) CNN-only/AUROC, (C) all models/nAUPRC, and (D) CNN-only/nAUPRC. Finally, while this work does not directly release a functional tool or a model, we follow and present the TRIPOD^41^ reporting guidelines for research focused on predictive models. This can be found in the Supplementary Section H.

## Ethics

This study was conducted using three publicly available, de-identified critical care datasets: MIMIC-IV, HiRID, and eICU. The MIMIC-IV database was approved by the institutional review boards (IRBs) of the Massachusetts Institute of Technology (MIT) and Beth Israel Deaconess Medical Center (BIDMC), and is made available under a data use agreement. All users are required to complete the CITI “Data or Specimens Only Research” course before accessing the data. The dataset is de-identified in accordance with the Health Insurance Portability and Accountability Act (HIPAA). The HiRID dataset was released with approval from the Ethics Commission of the Canton of Bern (KEK), Switzerland. The data are fully de-identified and were collected retrospectively from ICU patients at the Bern University Hospital. Informed consent was waived by the ethics committee due to the anonymized nature of the data. The eICU Collaborative Research Database is a multi-center critical care database developed by Philips Healthcare in collaboration with the MIT Laboratory for Computational Physiology. It is also de-identified in compliance with HIPAA and available under a data use agreement, requiring completion of the same CITI training course for access. Since all datasets used in this study are de-identified and publicly available, and the original data collection was approved by the respective ethics committees, no additional ethics approval was required for this secondary analysis.

## Supplementary information

Supplementary Information

## Data Availability

The three publicly available, de-identified datasets, HiRID, MIMIC-IV, and eICU, are available from Physionet after successful completion of the CITI “Data or Specimens Only Research” course. Harmonized datasets can be generated using the publicly available code provided in the YAIB repository.

## Code availability

The underlying code for this study is available in the GitLab repository https://gitlab.ethz.ch/BMDSlab/publications/sepsis/transfer-tearning-for-sepsis-detection “Transfer Learning For Sepsis Prediction”.
