# Supplementary material for: Evaluating deep learning sepsis prediction models in ICUs under distribution shift: a multi-centre retrospective cohort study
Source: NPJ Digit Med. 2026 Mar 3;9:306. doi: 10.1038/s41746-026-02364-4 (PMC13066616; doi:10.1038/s41746-026-02364-4)
Supplement: Supplementary file 1 — Supplementary Information [file 41746_2026_2364_MOESM1_ESM.pdf]

Supplementary Section A: Generalizability Across ICU Datasets and Relation Between Target Data Availability and AUROC and normalized AUPRC Performances.

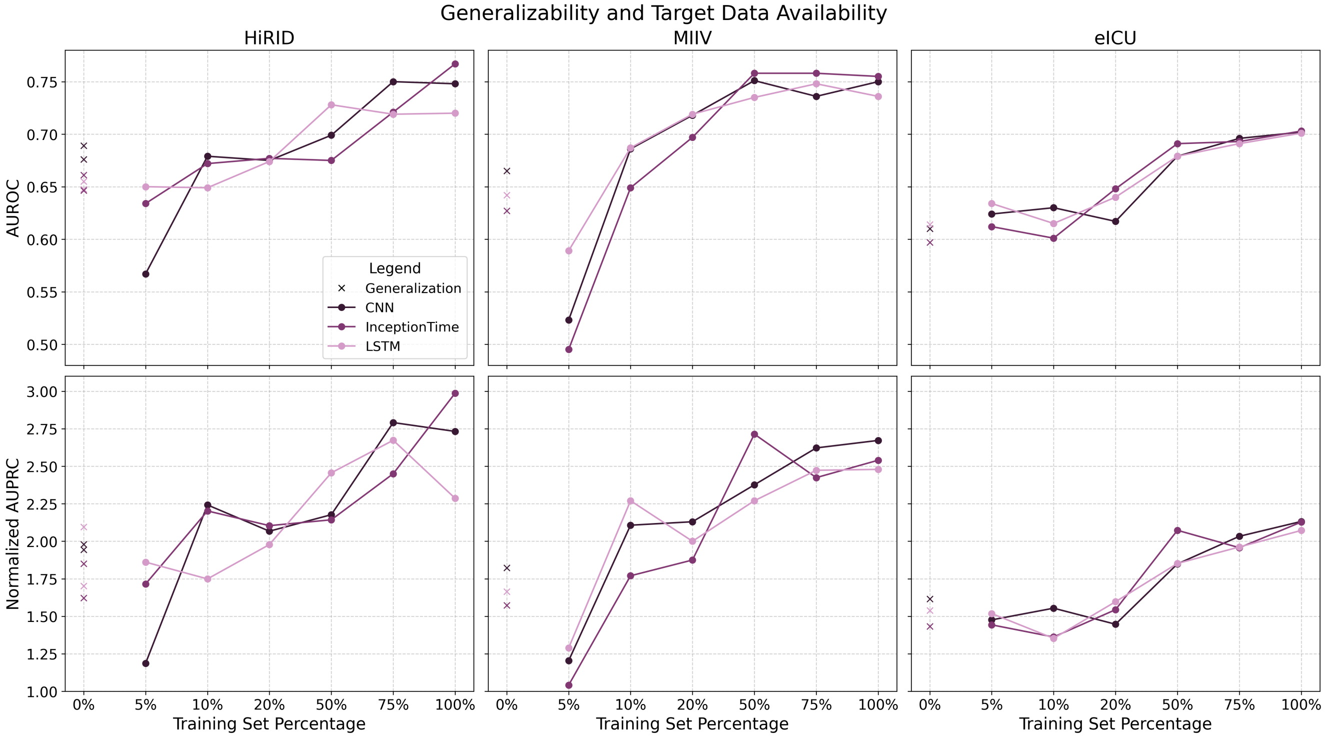

Supplementary Figure 1. Generalizability Across ICU Datasets and Relation Between Target Data Availability and AUROC and normalized AUPRC Performances. Three columns represent results for the three target domains HiRID, MIIV, and eICU. Generalization refers to how well models trained in source domains, performed for the target domain. The results for all CNN, InceptionTime, and LSTM are shown.

Supplementary Section B: Joint deep learning models' performances grouped by source-to-target combination.

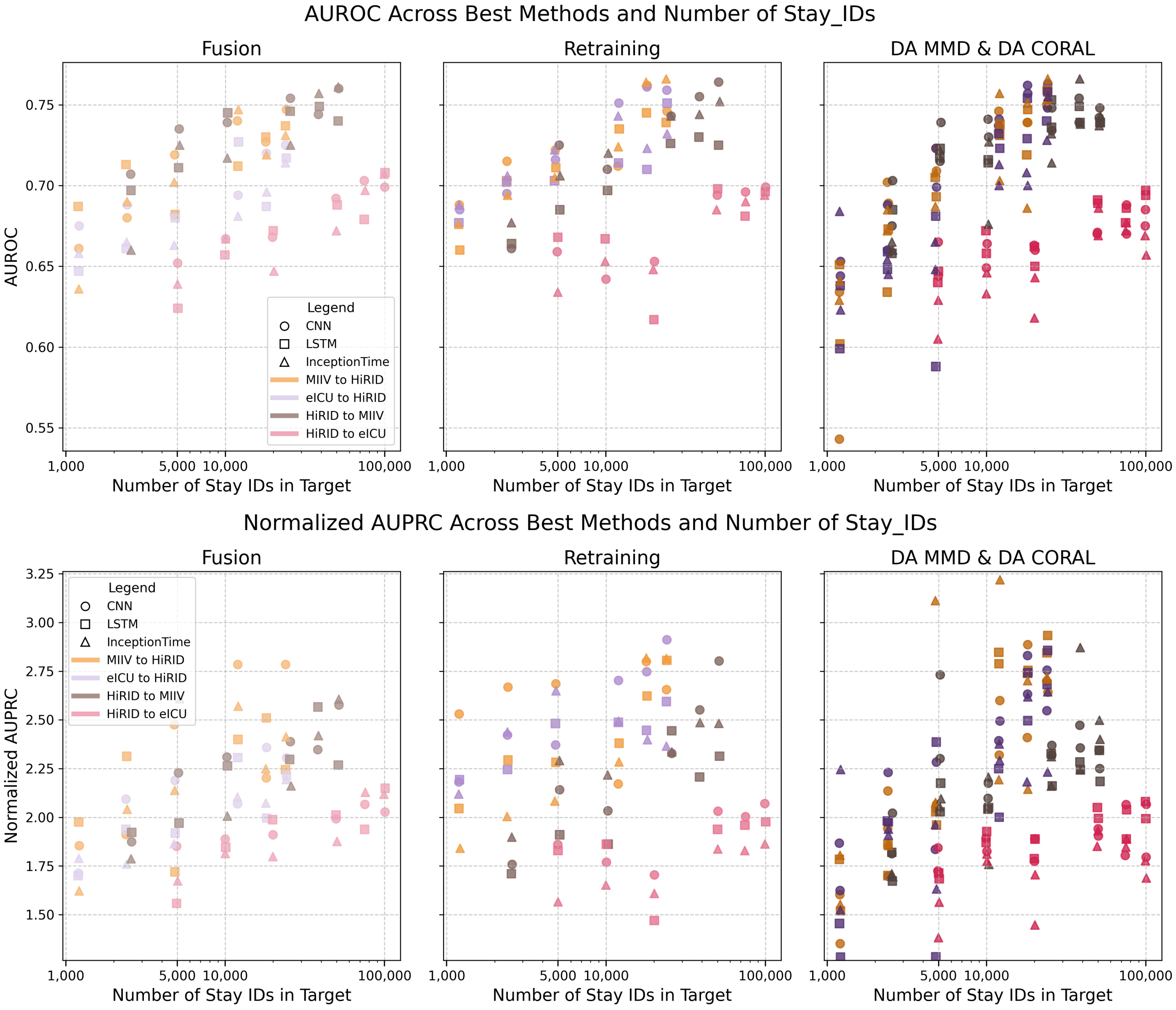

Supplementary Figure 2. Joint DL models' performances grouped by source-to-target combination, number of stay_ids within each training subset and transfer learning techniques.

Supplementary Section C: Deep learning models grouped by source-to-target combination, target dataset size, and transfer learning techniques.

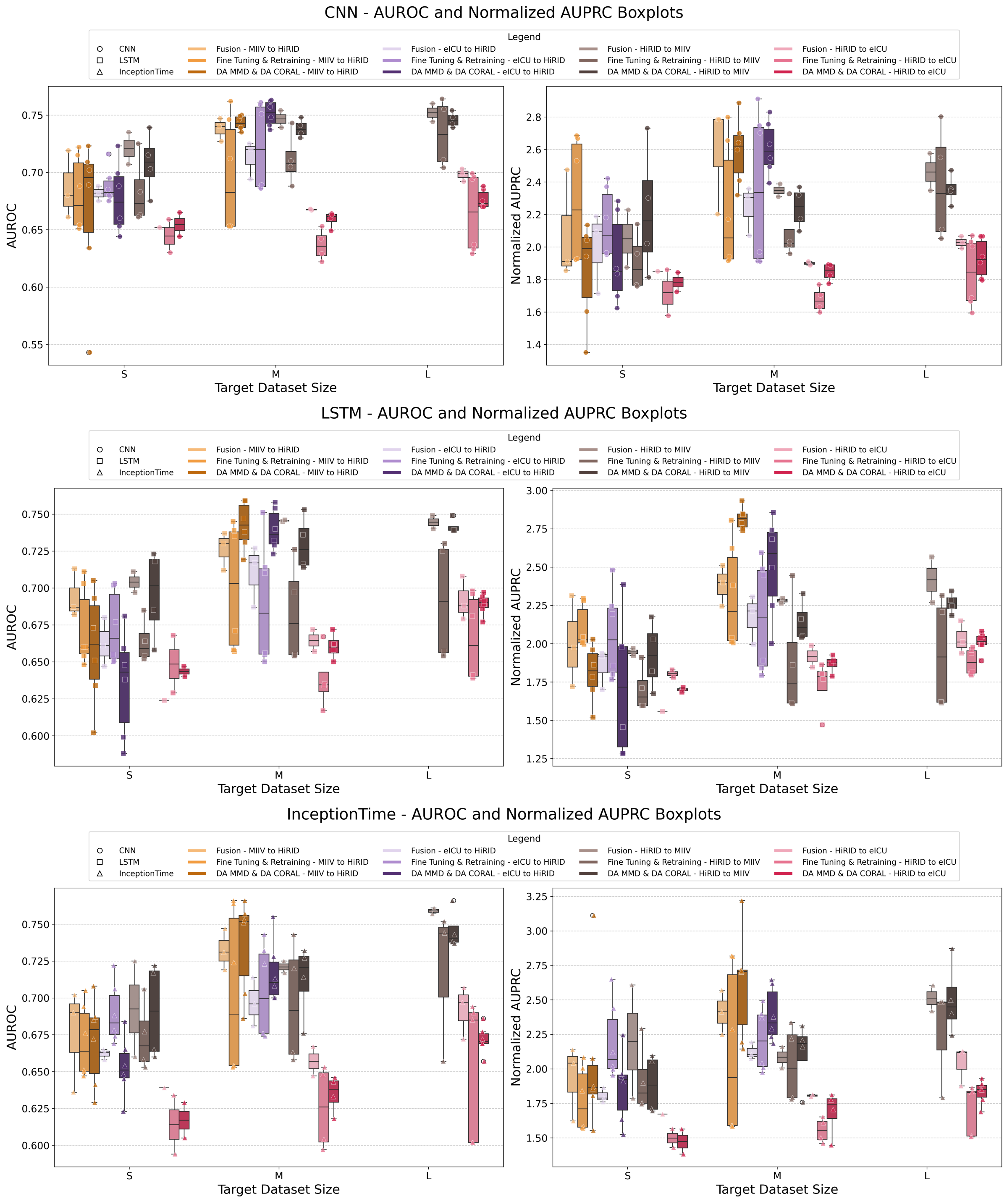

Supplementary Figure 3. DL models' performances grouped by source-to-target combination, target dataset size (small, medium and large) and transfer learning techniques.

Supplementary Section D: Core Results for Recall Metric

The main paper presents results for AUROC and normalized AUPRC. Shown here are the core figure results, as in the main paper, but for the recall metric.

Supplementary Table 1. Recall performance of models is evaluated from source to target. The deep learning model types include CNN, InceptionTime, and LSTM. Generalization refers to the performance of the source model on the target testing set. The percentages 5%, 10%, 20%, 50%, 75%, and 100% represent the performance achieved when that proportion of the training set is used to train a model (target training) and then evaluated on the target testing set.

			Target Training					
**Source to Target**	**Model**	**Generalization**	**5%**	**10%**	**20%**	**50%**	**75%**	**100%**
**MIIV to HiRID**	CNN	0.673	0.355	0.688	0.570	0.660	0.579	0.741
	InceptionTime	0.555	0.188	0.371	0.689	0.521	0.404	0.743
	LSTM	0.673	0.422	0.542	0.611	0.663	0.691	0.600
**eICU to HiRID**	CNN	0.826	0.355	0.688	0.570	0.660	0.579	0.741
	InceptionTime	0.798	0.188	0.371	0.689	0.521	0.404	0.743
	LSTM	0.653	0.422	0.542	0.611	0.663	0.691	0.600
**HiRID to MIIV**	CNN	0.456	0.033	0.677	0.626	0.721	0.692	0.712
	InceptionTime	0.390	0.164	0.359	0.684	0.597	0.765	0.568
	LSTM	0.306	0.496	0.442	0.665	0.726	0.684	0.715
**HiRID to eICU**	CNN	0.418	0.553	0.493	0.681	0.587	0.575	0.623
	InceptionTime	0.392	0.747	0.634	0.600	0.529	0.549	0.630
	LSTM	0.330	0.613	0.532	0.741	0.518	0.579	0.544

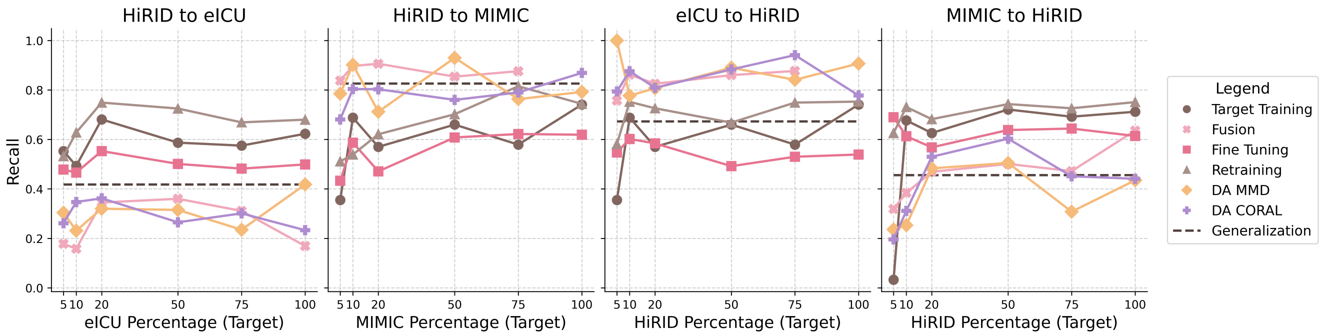

Supplementary Figure 4. Comparison of CNN model performance across different strategies, for different source to target pairs. Each subplot shows the Recall for the positive class as a function of the target dataset percentage used in the training process. The dashed brown line represents the Generalization baseline (performance of the source model in the target domain). Colored solid lines correspond to different training strategies, including Target Training, Fusion, Fine Tuning, Retraining, DA MMD, and DA CORAL.

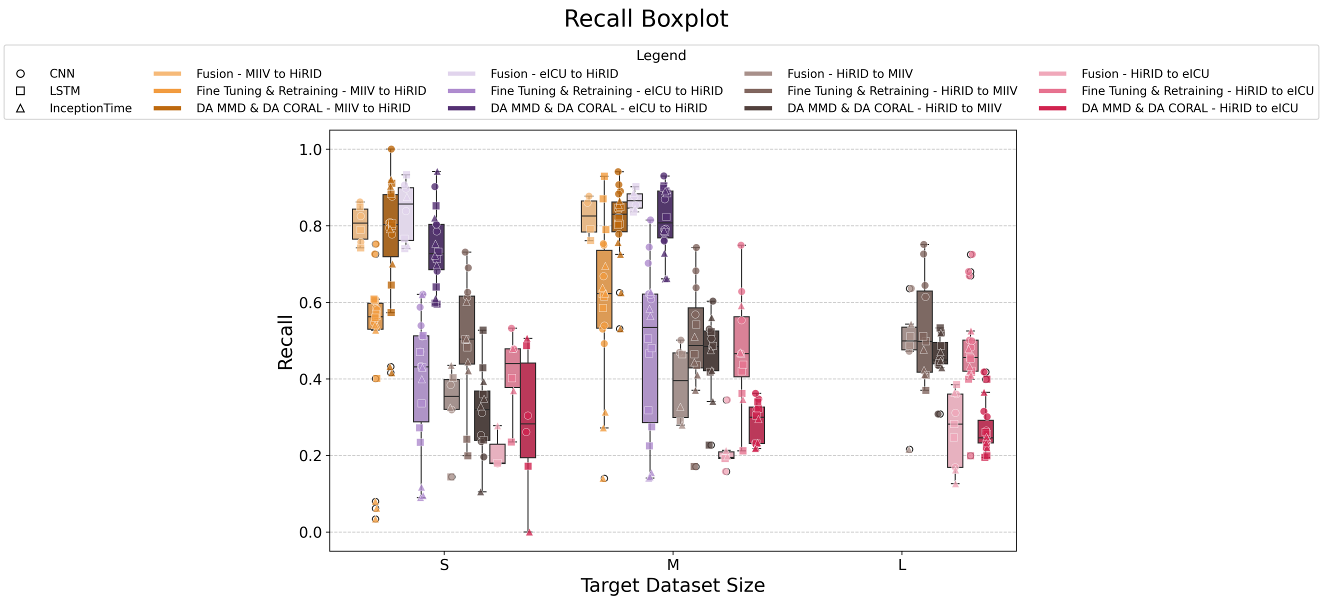

Supplementary Figure 5. Joint DL models' performances grouped by source-to-target combination, target dataset size (small, medium and large) and transfer learning techniques, for the recall metric.

4.6 Supplementary Section E: ICU Dataset Selected Features and Descriptions

Supplementary Table 2. ICU Dataset Selected Features and Descriptions

Feature	Abbreviation	Unit	Range
Blood pressure (systolic)	sbp	mmHg	60-250 mmHg
Blood pressure (diastolic)	dbp	mmHg	30-150 mmHg
Heart rate	hr	beats/minute	30-200 bpm
Mean arterial pressure	map	mmHg	40-160 mmHg
Oxygen saturation	o2sat	%	70-100%
Respiratory rate	resp	breaths/minute	6-40 breaths/minute
Temperature	temp	°C	33-42°C
Albumin	alb	g/dL	1.0-6.0 g/dL
Alkaline phosphatase	alp	IU/L	20-1200 IU/L
Alanine aminotransferase	alt	IU/L	5-2000 IU/L
Aspartate aminotransferase	ast	IU/L	5-2000 IU/L
Base excess	be	mmol/L	-20 to +20 mmol/L
Bicarbonate	bicar	mmol/L	10-40 mmol/L
Bilirubin (total)	bili	mg/dL	0.1-30 mg/dL
Bilirubin (direct)	bili_dir	mg/dL	0-10 mg/dL
Band form neutrophils	bnd	%	0-20%
Blood urea nitrogen	bun	mg/dL	5-150 mg/dL
Calcium	ca	mg/dL	6-14 mg/dL
Calcium ionized	cai	mmol/L	0.625-1.75 mmol/L
Creatinine	crea	mg/dL	0.2-15 mg/dL
Creatine kinase	ck	IU/L	20-20,000 IU/L
Creatine kinase MB	ckmb	ng/mL	0-50 ng/mL
Chloride	cl	mmol/L	80-130 mmol/L
CO2 partial pressure	pco2	mmHg	20-70 mmHg
C-reactive protein	crp	mg/L	0-300 mg/L
Fibrinogen	fgn	mg/dL	100-700 mg/dL
Glucose	glu	mg/dL	20-600 mg/dL
Haemoglobin	hgb	g/dL	5-20 g/dL
International normalised ratio (INR)	inr_pt	-	0.5-5.0
Lactate	lact	mmol/L	0.5-15 mmol/L
Lymphocytes	lymph	%	0-80%
Mean cell haemoglobin	mch	pg	20-40 pg/cell
Mean corpuscular haemoglobin concentration	mchc	%	25-40%
Mean corpuscular volume	mcv	fL	60-120 fL
Methaemoglobin	methb	%	0-20%
Magnesium	mg	mg/dL	1.0-4.0 mg/dL
Neutrophils	neut	%	0-90%
O2 partial pressure	po2	mmHg	40-200 mmHg
Partial thromboplastin time	ptt	sec	20-100 seconds
pH of blood	ph	-	6.8-7.8

Continued on next page

Supplementary Table 2. – continued from previous page

**Feature**	**Abbreviation**	**Unit**	**Range**
Phosphate	phos	mg/dL	1.0-7.0 mg/dL
Platelets	plt	1,000/μL	1,000-100,000/μL
Potassium	k	mmol/L	2.5-7.0 mmol/L
Sodium	na	mmol/L	120-160 mmol/L
Troponin T	tnt	ng/mL	0-10 ng/mL
White blood cells	wbc	1,000/μL	1,000-100,000/μL
Fraction of inspired oxygen	fio2	%	21-100%
Urine output	urine	mL	0-5000 mL/day

4.7 Supplementary Section F: Sepsis Sample Prevalence Across Training Sets

Supplementary Table 3. Sepsis Sample Prevalences Across Training Sets of the Target Domain: Percentage of samples which have a sepsis positive label, for each dataset and size combination. These prevalences are after pre-processing and windowing, therefore representing the number of positive samples that the model sees during training.

Dataset	Sub-Sampled Dataset Size					
	**5%**	**10%**	**20%**	**50%**	**75%**	**100%**
eICU	3.38%	3.39%	2.71%	3.39%	3.45%	3.38%
MIIV	3.11%	3.35%	3.47%	3.56%	3.63%	3.68%
HiRID	5.84%	5.51%	5.60%	5.59%	5.51%	5.63%

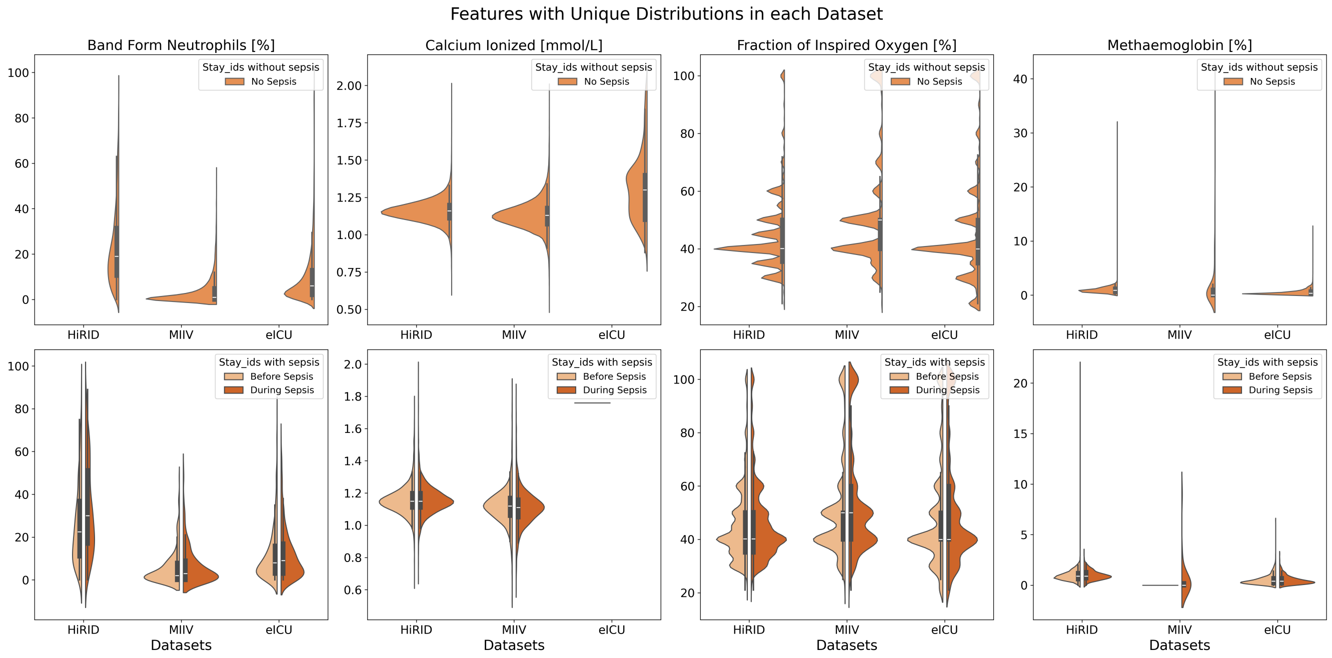

Supplementary Figure 6. Features with Unique Distributions in each Dataset.

4.8 Supplementary Section G: Feature Distributions

To examine the intricacies of distribution shifts in ICU data, we investigated distribution patterns for several dynamic features, visualizing them through violin plots and stratifying by the outcome of sepsis. For patients who developed sepsis, we also examined measurements before and during the onset of sepsis to see how the distribution might evolve over these time periods. A closer look at these dynamic features through violin plots revealed three key insights about dataset-specific behaviors. First, certain variables showed unique distributions in each dataset (Figure 13): *bnd* (band form neutrophils) and *cai* (ionized calcium) spanned a wider range in HiRID than in MIIV or eICU, while *cai* and *methb* (methemoglobin) were rarely documented in American data sets for patients with sepsis. Furthermore, *fio2* (fraction of inspired oxygen) exhibited multiple distinct peaks whose concentration levels varied between the three cohorts. In Figure 14, other features: *alb* (albumin), *cl* (chloride), *dbp* (diastolic blood pressure), and *mchc* (mean corpuscular hemoglobin concentration), showed a marked shift in the HiRID data set relative to MIIV and eICU, persisting in both sepsis subpopulations. Finally, as shown in Figure 15, *C-reactive protein* displayed a notably wider distribution in the eICU compared to the other two datasets, an observation consistent between the sepsis and non-sepsis groups. These varied distribution patterns highlight the substantial heterogeneity between hospitals that can undermine naive data pooling or model deployment without adaptation, as local definitions, measurement ranges, and clinical protocols for critical laboratory values appear to differ significantly between these ICU settings.

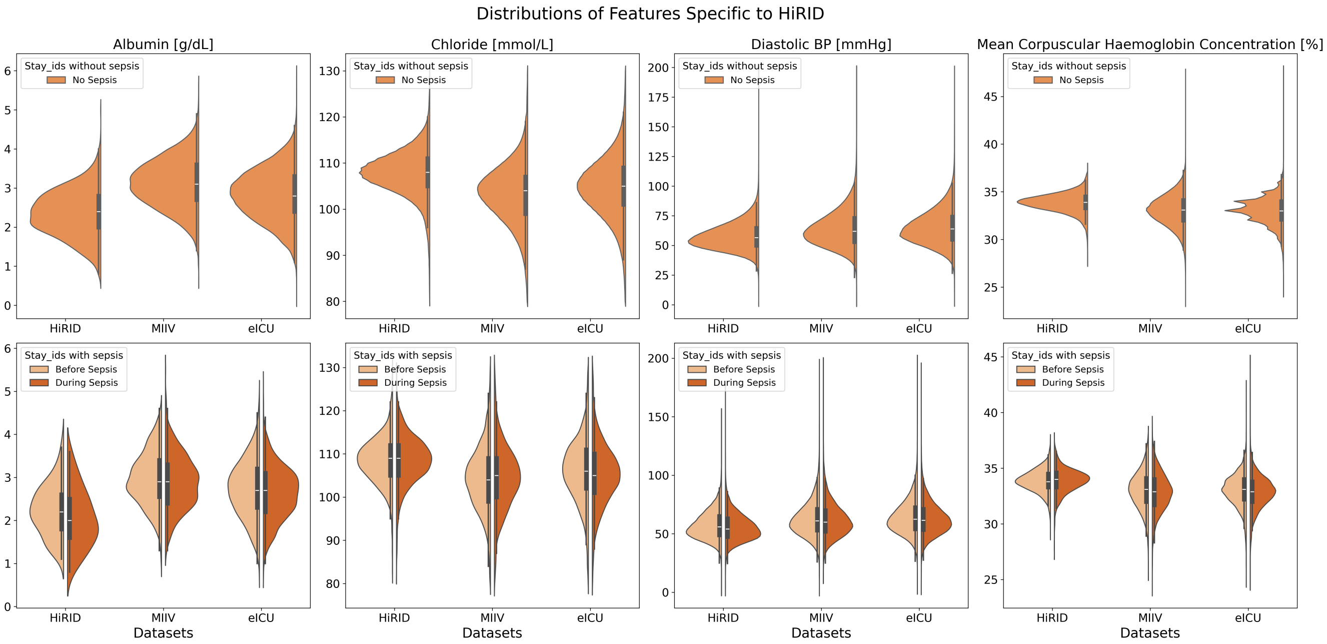

Supplementary Figure 7. Features with Distributions Specific to HiRID Dataset.

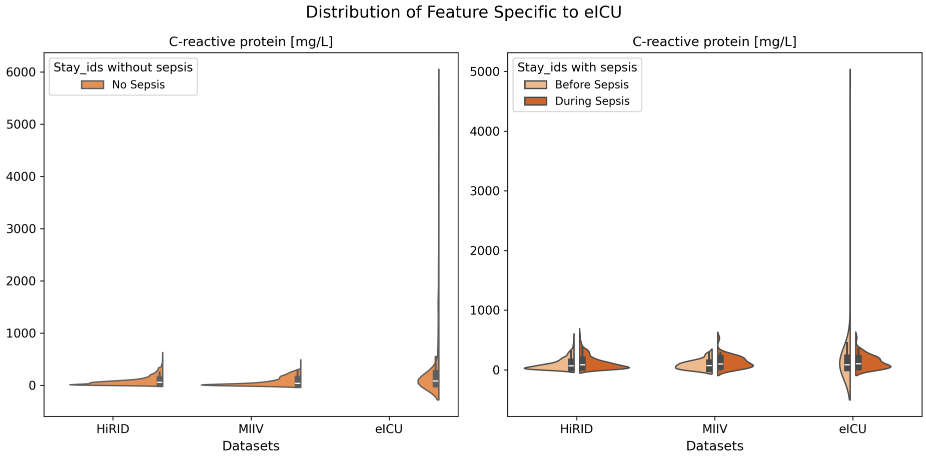

Supplementary Figure 8. C-Reactive Protein Distribution Specific to eICU Dataset.

Supplementary Section H: TRIPOD Reporting Checklist

While this work does not directly release a prediction model, we nevertheless report a modified version of the TRIPOD+AI[42] checklist for prediction model studies, for full visiblity and transparency.

Supplementary Table 4. TRIPOD+AI Checklist for Prediction Model Studies

Section/Topic	Item	D/E[1]	Checklist item	Page
**INTRODUCTION**				
Background	3a	D;E	Explain the healthcare context (including whether diagnostic or prognostic) and rationale for developing or evaluating the prediction model, including references to existing models	2
	3b	D;E	Describe the target population and the intended purpose of the prediction model in the context of the care pathway, including its intended users (e.g., healthcare professionals, patients, public)	2
	3c	D;E	Describe any known health inequalities between sociodemographic groups	13
Objectives	4	D;E	Specify the study objectives, including whether the study describes the development or validation of a prediction model (or both)	2
**METHODS**				
Data	5a	D;E	Describe the sources of data separately for the development and evaluation datasets (e.g., randomised trial, cohort, routine care or registry data), the rationale for using these data, and representativeness of the data	14
	5b	D;E	Specify the dates of the collected participant data, including start and end of participant accrual; and, if applicable, end of follow-up	14
Participants	6a	D;E	Specify key elements of the study setting (e.g., primary care, secondary care, general population) including the number and location of centres	14
	6b	D;E	Describe the eligibility criteria for study participants	15
	6c	D;E	Give details of any treatments received, and how they were handled during model development or evaluation, if relevant	N/A
Data preparation	7	D;E	Describe any data pre-processing and quality checking, including whether this was similar across relevant sociodemographic groups	15
Outcome	8a	D;E	Clearly define the outcome that is being predicted and the time horizon, including how and when assessed, the rationale for choosing this outcome, and whether the method of outcome assessment is consistent across sociodemographic groups	15
	8b	D;E	If outcome assessment requires subjective interpretation, describe the qualifications and demographic characteristics of the outcome assessors	N/A
	8c	D;E	Report any actions to blind assessment of the outcome to be predicted	N/A

Continued on next page

Table 10 – *Continued from previous page*

Section/Topic	Item	D/E[1]	Checklist item	Page
Predictors	9a	D	Describe the choice of initial predictors (e.g., literature, previous models, all available predictors) and any pre-selection of predictors before model building	14
	9b	D;E	Clearly define all predictors, including how and when they were measured (and any actions to blind assessment of predictors for the outcome and other predictors)	14
	9c	D;E	If predictor measurement requires subjective interpretation, describe the qualifications and demographic characteristics of the predictor assessors	N/A
Sample size	10	D;E	Explain how the study size was arrived at (separately for development and evaluation), and justify that the study size was sufficient to answer the research question. Include details of any sample size calculation	15
Missing data	11	D;E	Describe how missing data were handled. Provide reasons for omitting any data	15
Analytical methods	12a	D	Describe how the data were used (e.g., for development and evaluation of model performance) in the analysis, including whether the data were partitioned, considering any sample size requirements	17-18
	12b	D	Depending on the type of model, describe how predictors were handled in the analyses (functional form, rescaling, transformation, or any standardisation)	18
	12c	D	Specify the type of model, rationale[2], all model-building steps, including any hyperparameter tuning, and method for internal validation	18
	12d	D;E	Describe if and how any heterogeneity in estimates of model parameter values and model performance was handled and quantified across clusters (e.g., hospitals, countries). See TRIPOD-Cluster for additional considerations[3]	19
	12e	D;E	Specify all measures and plots used (and their rationale) to evaluate model performance (e.g., discrimination, calibration, clinical utility) and, if relevant, to compare multiple models	16
	12f	E	Describe any model updating (e.g., recalibration) arising from the model evaluation, either overall or for particular sociodemographic groups or settings	N/A
	12g	E	For model evaluation, describe how the model predictions were calculated (e.g., formula, code, object, application programming interface)	18
Class imbalance	13	D;E	If class imbalance methods were used, state why and how this was done, and any subsequent methods to recalibrate the model or the model predictions	18-19
Fairness	14	D;E	Describe any approaches that were used to address model fairness and their rationale	N/A

Continued on next page

Table 10 – *Continued from previous page*

Section/Topic	Item	D/E[1]	Checklist item	Page
Model output	15	D	Specify the output of the prediction model (e.g., probabilities, classification). Provide details and rationale for any classification and how the thresholds were identified	17
Training versus evaluation	16	D;E	Identify any differences between the development and evaluation data in healthcare setting, eligibility criteria, outcome, and predictors	N/A
Ethical approval	17	D;E	Name the institutional research board or ethics committee that approved the study and describe the participant-informed consent or the ethics committee waiver of informed consent	19
**OPEN SCIENCE**				
Funding	18a	D;E	Give the source of funding and the role of the funders for the present study	19-20
Conflicts of interest	18b	D;E	Declare any conflicts of interest and financial disclosures for all authors	19-20
Protocol	18c	D;E	Indicate where the study protocol can be accessed or state that a protocol was not prepared	19-20
Registration	18d	D;E	Provide registration information for the study, including register name and registration number, or state that the study was not registered	
Data sharing	18e	D;E	Provide details of the availability of the study data	
Code sharing	18f	D;E	Provide details of the availability of the analytical code[4]	19
**PATIENT & PUBLIC INVOLVEMENT**				
Patient & Public Involvement	19	D;E	Provide details of any patient and public involvement during the design, conduct, reporting, interpretation, or dissemination of the study or state no involvement	N/A
**RESULTS**				
Participants	20a	D;E	Describe the flow of participants through the study, including the number of participants with and without the outcome and, if applicable, a summary of the follow-up time. A diagram may be helpful	3,15
	20b	D;E	Report the characteristics overall and, where applicable, for each data source or setting, including the key dates, key predictors (including demographics), treatments received, sample size, number of outcome events, follow-up time, and amount of missing data. A table may be helpful. Report any differences across key demographic groups	14, Supp.
	20c	E	For model evaluation, show a comparison with the development data of the distribution of important predictors (demographics, predictors, and outcome)	N/A

Continued on next page

Table 10 – *Continued from previous page*

Section/Topic	Item	D/E[1]	Checklist item	Page
Model development	21	D;E	Specify the number of participants and outcome events in each analysis (e.g., for model development, hyperparameter tuning, model evaluation)	15
Model specification	22	D	Provide details of the full prediction model (e.g., formula, code, object, application programming interface) to allow predictions in new individuals and to enable third-party evaluation and implementation, including any restrictions to access or re-use (e.g., freely available, proprietary)[5]	20
Model performance	23a	D;E	Report model performance estimates with confidence intervals, including for any key subgroups (e.g., sociodemographic). Consider plots to aid presentation	11
	23b	D;E	If examined, report results of any heterogeneity in model performance across clusters. See TRIPOD-Cluster for additional details[3]	10-11
Model updating	24	E	Report the results from any model updating, including the updated model and subsequent performance	N/A
**DISCUSSION**				
Interpretation	25	D;E	Give an overall interpretation of the main results, including issues of fairness in the context of the objectives and previous studies	10-11
Limitations	26	D;E	Discuss any limitations of the study (such as a non-representative sample, sample size, overfitting, missing data) and their effects on any biases, statistical uncertainty, and generalizability	13-14
Usability of the model in the context of current care	27a	D	Describe how poor quality or unavailable input data (e.g., predictor values) should be assessed and handled when implementing the prediction model	13-14
	27b	D	Specify whether users will be required to interact in the handling of the input data or use of the model, and what level of expertise is required of users	N/A
	27c	D;E	Discuss any next steps for future research, with a specific view to applicability and generalizability of the model	13-14

[1]D = items relevant only to the development of a prediction model; E = items relating solely to the evaluation of a prediction model; D;E = items applicable to both the development and evaluation of a prediction model.
[2]Separately for all model building approaches.
[3]TRIPOD-Cluster is a checklist of reporting recommendations for studies developing or validating models that explicitly account for clustering or explore heterogeneity in model performance (e.g., at different hospitals or centres). Debray et al, BMJ 2023; 380: e071018 [DOI: 10.1136/bmj-2022-071018].
[4]This relates to the analysis code, for example, any data cleaning, feature engineering, model building, evaluation.
[5]This relates to the code to implement the model to get estimates of risk for a new individual.

From: Collins GS, Moons KGM, Dhiman P, et al. BMJ 2024;385:e078378. doi:10.1136/bmj-2023-078378

Supplementary Section I: Data Harmonization and Pre-Processing Summary.

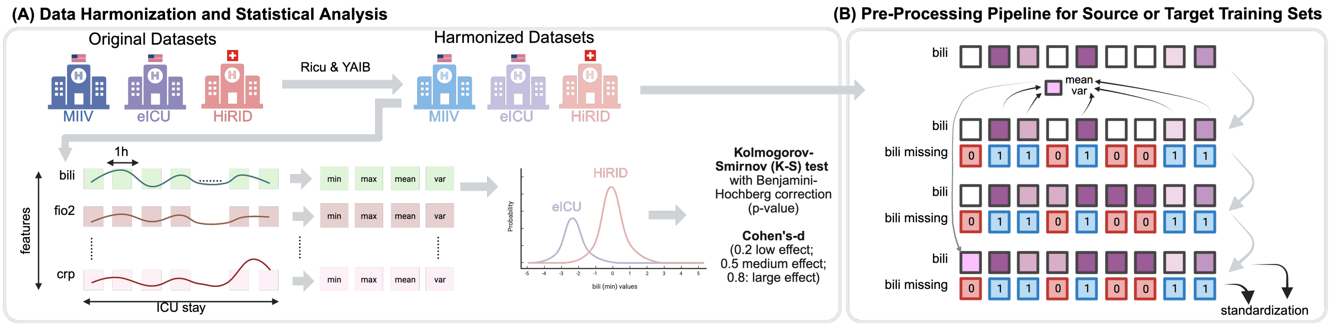

Supplementary Figure 9. An overview of data harmonization and pre-processing: **(A)** Original datasets were processed using the RiCU and YAIB pipelines to generate harmonized datasets that allow like-to-like comparisons between the datasets at the feature level. Using these harmonized datasets, we obtained basic statistical properties of patient trajectories, such as minimum, maximum, mean, and variance, at the feature level. These statistical measures were then compared across MIIV-eICU, MIIV-HiRID, and eICU-HiRID combinations using the Kolmogorov-Smirnov (K-S) test and Cohen's *d*; **(B)** Shows the data pre-processing pipeline used to prepare harmonized data for model training. An example is provided for the feature "bili," which contains some missing data. Initially, using the available data, the mean and variance are calculated. Then, missing flags are generated as an additional feature column. Next, features are forward-filled, after which any remaining missing data are filled with the mean calculated earlier. Afterwards, all features are standardized. Values learned during this process are applied to both training and validation sets;
